# Interaction between bacterial phytochromes Agp1 and Agp2 of *Agrobacterium fabrum* by fluorescence resonance energy transfer and docking studies

**DOI:** 10.1002/1873-3468.15102

**Published:** 2025-01-26

**Authors:** Afaf El Kurdi, Gero Kaeser, Patrick Scheerer, David Hoffmann, Ebru Akkus, Marcus Elstner, Norbert Krauß, Tilman Lamparter

**Affiliations:** ^1^ Allgemeine Botanik Karlsruhe Institute of Technology, Joseph Kölreuter Institut für Pflanzenwissenschaften (JKIP) Karlsruhe Germany; ^2^ Charité ‐ Universitätsmedizin Berlin, Institute of Medical Physics and Biophysics, Group Structural Biology of Cellular Signaling Berlin Germany; ^3^ Institut für Physikalische Chemie Karlsruhe Institute of Technology Karlsruhe Germany

**Keywords:** biliprotein, DNA transfer, histidine kinase, molecular docking, photoreceptor, protein interaction

## Abstract

Phytochromes are biliprotein photoreceptors found in bacteria, fungi, and plants. The soil bacterium *Agrobacterium fabrum* has two phytochromes, Agp1 and Agp2, which work together to control DNA transfer to plants and bacterial conjugation. Both phytochromes interact as homodimeric proteins. For fluorescence resonance energy transfer (FRET) measurements, various Agp1 mutants and wild‐type Agp2 were labeled with specific fluorophores to study their interaction. FRET efficiencies rose from position 122 to 545 of Agp1. The photosensory chromophore module (PCM) of Agp1 did not show a FRET signal, but the PCM of Agp2 did. Docking models suggest that Agp1 and Agp2 interact with their histidine kinase and PCM perpendicular to each, around 45 amino acids of Agp1 or Agp2 are involved.

## Graphical abstract

The interaction between *Agrobacterium fabrum* phytochromes Agp1 and Agp2 was studied using FRET. Full‐length and truncated proteins with varied label positions provided spatial insights, narrowing down from many docking models to three. These models explain Agp2's reduction in Agp1 autophosphorylation, with Arg83 of Agp2 specifically evolved to interact with Agp1, unlike Agp2 in species without Agp1.

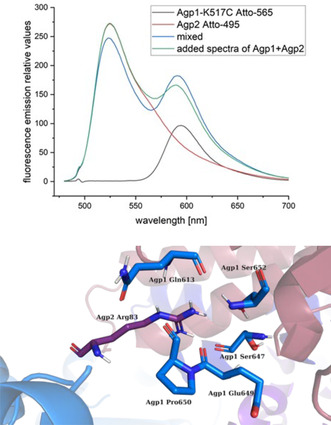

## Abbreviations

***E***_**FRET**_, energy transfer in FRET

**FRET**, fluorescence resonance energy transfer

**GAF domain**, domain of cGMP specific phosphodiesterase, adenylate cyclase, FhlA

**PAS domain**, domain in period, Arndt, simple minded

**PCM**, photosensory chromophore module

Phytochromes are photoreceptor proteins with a bilin chromophore that undergo light triggered conversions between the two spectrally distinct forms, Pr and Pfr. The model bacterium *Agrobacterium fabrum* contains two phytochromes termed Agp1 and Agp2. In darkness, Agp1 adopts the Pr form with a maximum absorbance around 700 nm, whereas the bathy phytochrome Agp2 adopts the Pfr form with a maximum absorbance around 750 nm [1]. Canonical phytochromes, including Agp1 and Agp2, carry three characteristic domains, the PAS, GAF, and PHY domains, in the N terminus. The C terminus of Agp1 and Agp2, and of most other bacterial and of fungal phytochromes, contains a histidine kinase. Agp2 has an additional response regulator at its C terminus, a domain that can act as phospho‐receiver of the histidine kinase. The domain structures of other bacterial phytochromes, plant phytochromes, and fungal phytochromes are similar. Plant phytochromes have additional two PAS domains between the PCM and the C‐terminal histidine kinase module. Crystal structures of PCM and shorter fragments of phytochromes show that the PAS and GAF domains form an exceptional knotted structure and that a characteristic tongue‐like structure folds back from the PHY domain onto the chromophore pocket of the GAF domain [2, 3, 4, 5]. Bacterial phytochrome dimers with histidine kinases have a parallel head to head arrangement in which the histidine kinase form strong dimerization units [6, 7, 8, 9], whereas plant phytochromes adopt a completely different, more complex quaternary dimer structure with asymmetric subunits [10].

The Pr and Pfr forms of phytochromes are stable or semistable, spectrally characteristic forms, the names stand for red light absorbing and far‐red light absorbing pigment. Most bacterial phytochromes including Agp1 and Agp2 incorporate biliverdin (BV) as chromophore, which is attached to a Cys close to the N terminus. Photoconversion from Pr to Pfr and from Pfr to Pr are initiated by *Z‐E* and *E‐Z* isomerizations around the C15–C16 methine bridge of the bilin [11]. This isomerization triggers conformational changes in the protein via several intermediates that are reflected by spectral changes. Major structural changes during photoconversion are found in the tongue region of the PHY domain, in which transition of ß‐sheet structure to α‐helical structure is observed [12, 13, 14].

Agp1 and Agp2 control conjugation and the gene transfer to plants in *A. fabrum* [15, 16], possibly by interaction with VirD2 and TraA [17]. *Agrobacterium agp1* ^−^ and *agp2* ^−^ knockout mutants have a reduced conjugation rate, and the double knockout has no conjugation. A similar pattern was observed for gene transfer into plants. Both DNA transfer events are also downregulated in the light. Because Agp1 and Agp2 act together in both effects, we suggested that the proteins might interact with each other. Indeed, this interaction between Agp1 and Agp2 was confirmed by assays on spectral properties, by quantification of Agp1 and Agp2 phosphorylation, and by fluorescence resonance energy transfer (FRET) [18].

It is generally assumed that phytochromes adopt a homodimer quaternary structure. For Agp1, tight homodimer formation was shown by size exclusion chromatography, crosslinking and proteolysis [19], and dimer formation of Agp2 was shown by size exclusion chromatography [18]. It is therefore likely that an interaction of Agp1 and Agp2 occurs between two homodimers.

**Fig. 1:**
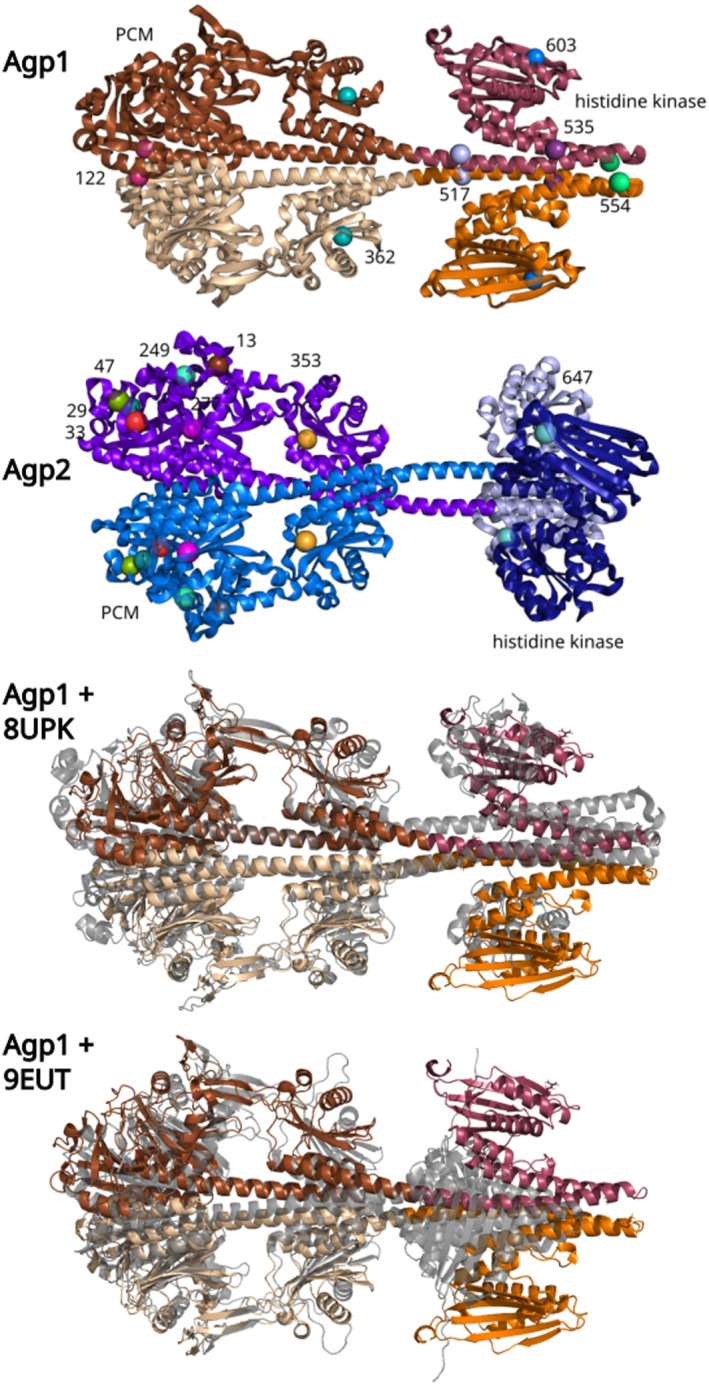
Agp1 and Agp2 full length dimer models. The models were generated by alphafold 2, showing a high similarity with crystal structures for monomers in the N terminus and with related histidine kinase monomers in the C terminus. The spheres in the upper two panels indicate the positions of the Atto labels. The numbers next to the spheres show the amino acid positions of one monomer. The same amino acid on the other monomer is drawn in the same color. The lower two panels show the structure of Agp1 aligned with the Cryo EM structure of *Stigmatella auriantaca* phytochrome (PDB ID: 8UPK) and *Pseudomonas aeruginosa* phytochrome (PDB ID: 9EUT). Agp1 is drawn in the same colors as above, the Cryo EM structures are drawn in gray. Coloring of Agp1 and Agp2 is also comparable with Fig. S1.

FRET is based on distance‐dependent energy transfer between two different fluorescent dyes. The donor and acceptor fluorophore pair must be selected in a way that donor emission and acceptor excitation spectra do overlap [20]. We expanded our FRET interaction studies with Agp1 and Agp2 by using different mutants of Agp1, which were labeled at Positions 122, 362, 517, 535, 554, and 603. Agp2 was used as a wild‐type protein, and it has seven cysteines at Positions 29, 33, 47, 249, 277, 353, and 647, which could function as anchor sites for fluorescent labels (Fig. 1). Multiple labels make the analysis more difficult, but with the aid of modeling, it was possible to get a detailed impression about the relative orientations of the interacting Agp1 and Agp2 homodimers to each other.

## Materials and methods

### Extinction coefficients and Förster‐radius

We used the following extinction coefficients (ε) for estimation of concentrations from spectral measurements. The 280 nm values are estimated from the number of Tyr and Trp residues: Agp1 apoprotein at 280 nm: ε = 100 000 m ^−1^·cm^−1^ [21]; Agp1‐PCM apoprotein at 280 nm: ε = 78 400 m ^−1^·cm^−1^ [21]; Agp1 Pr holoprotein at 700 nm: ε = 90 000 m ^−1^·cm^−1^ [22]; Agp2 apoprotein at 280 nm: ε = 81 300 m ^−1^·cm^−1^ [21]; Agp2 PCM apoprotein at 280 nm: ε = 57 900 m ^−1^·cm^−1^; Agp2 histidine kinase and response regulator: 23 600 m ^−1^·cm^−1^; Agp2 Pfr holoprotein at 750 nm: ε = 45 000 m ^−1^·cm^−1^ (estimated from running experiments); free BV at 696 nm in methanol/HCl: ε = 30 800 m ^−1^·cm^−1^ [23]; free Atto‐495 at 495 nm: ε = 80 000 m ^−1^·cm^−1^; free Atto‐565 at 565 nm: ε = 120 000 m ^−1^·cm^−1^ (both from manufacturer). Upon incorporation into the protein, Atto‐495 can split into two spectral species with absorption maxima at ca. 500 nm and ca. 475 nm. Only the 500 nm species is fluorescent. The extinction coefficient of Atto‐495 can decrease upon incorporation into the protein. The radius of half maximal energy transfer, the Förster Radius, between Atto‐495 and Atto‐565 is *R* = 50 Å, according to the information given by the manufacturer. We did not consider the minor contribution of chromophore or fluorophores to the absorbance at 280 nm of the protein.

### Recombinant phytochromes and mutants

Note that in databases and in the literature, two versions of Agp1 numbering can exist, due to two different start codons. The expression construct used here is based on a sequence with nine more N‐terminal amino acids than the sequence now present in protein databases. We used six mutants of full‐length *A. fabrum* phytochrome Agp1, A122C, A362C, K517C, R535C, K554C, and R603C, which have a Cys at the given position and the chromophore binding Cys 20. The other two Agp1 wild‐type cysteines were modified to alanine or serine. The mutant A362C was also used as PCM version (N terminal PAS, GAF and PHY domains, lacking the histidine kinase). The Agp1 mutants are also described in earlier work [24, 25]. The full length wild‐type *A. fabrum* phytochrome Agp2 was labeled through its natural cysteines: The chromophore binding cysteine is at Position 13, the other cysteines are at Positions 29, 33, 47, 249, 277, 353, and 647. We generated the mutants C47S, C249S, C277S, and C353S of Agp2 by site‐directed mutagenesis [26] for side tests. A new expression vector for the histidine kinase of Agp2 was obtained by PCR with primers GTGCTCGAGTGCGGCCGCAA/TTGATGGCAGGCGAAAGAGAGCG using pAg2 as template, which was transformed into *Escherichia coli* DH5‐alpha after recircularization.

### Protein expression and purification

Agp1, Agp2, and mutants thereof were expressed using *E. coli* DH5‐alpha strains with pET21b‐based expression vectors pAg1 and pAg2 [24, 25, 27, 28]. Purifications occurred as apoproteins with no chromophore addition during purification. All expression vectors encode for proteins with a C‐terminal 6‐his tag. After growth of *E. coli* at the desired temperature and IPTG induction of specific expression, proteins were extracted with a French Press. Supernatant of a centrifugation was precipitated using 50% of ammonium sulfate for full length proteins and 66% ammonium sulfate for the shorter protein fragments Agp1A362C‐PCM and Agp2‐PCM. Affinity purification was performed using a nickel‐agarose column to which the 6‐His tag binds. Binding to the column and washing were performed with 50 mm Tris/Cl, 300 mm NaCl, and 10 mm imidazole pH 7.8 buffer. For elution, 50 mm Tris/Cl, 300 mm NaCl, and 250 mm imidazole pH 7.8 buffer was used. Fractions containing protein were collected and subjected to ammonium sulfate precipitation. The pellet was finally dissolved in 50 mm Tris/Cl, 5 mm EDTA, 300 mm NaCl, pH 7.8. Purifications are also described in earlier publications [3, 29, 30]. Agp1 and Agp2 were essentially pure after purification according to SDS/PAGE results, showing one band on the gel. Protein concentrations were determined by measuring A_280 nm_. The final concentration of the proteins were between 20 and 40 μm.

### Biliverdin assembly

Biliverdin (BV, Sigma‐Aldrich, Munich, Germany) was prepared by loading a ca. 1 mL 5 mm solution in aqueous solution to 1 mL Sep‐Pak C18 cartridges (Waters, Milford, MA, USA). The column was washed with water and air. BV was eluted with methanol and concentrated in a spin vacuum at 4 °C and finally dissolved in DMSO at 10 mm and stored at −80 °C for further experiments. BV concentrations were obtained by measuring A_696 nm_ in methanol/HCl.

The assembly with BV and labeling with Atto dye were performed in darkness or under green LED safety light. The apoproteins had a start concentration of 10–15 μm as determined by OD_280 nm_, the incubation buffer was 50 mm Tris/Cl, 5 mm EDTA, 300 mm NaCl, pH 7.8.

The apoprotein was reduced with 1 mm TCEP from a 100 mm stock solution. After 30 min at RT or longer, BV was added to a threefold molar excess and the mixture was incubated for 2 h at RT. The assembly was followed by UV/vis absorbance spectroscopy. Excess BV and TCEP were separated from the protein by NAP 5 columns (GE healthcare, Munich, Germany) according to the instructions of the manufacturer.

### Fluorophore labeling

The selected fluorophores (Atto dyes, Atto‐Tec, Siegen, Germany) had a reactive maleimide group that can bind covalently to the thiol group of a cysteine. Atto‐565 and Atto‐495 fluorophore stock solutions were prepared at concentrations of 10 mm in DMSO and stored at −80 °C. The final Atto concentration for labeling of Agp1 was 16 μm, and the protein concentration was 8 μm.

In case of Agp2, the Atto‐495 concentration was 8 μm and the protein concentration also 8 μm. The incorporated fluorophore revealed a dual‐band absorbance with peaks at 475 nm and at 500 nm. Such a band splitting effect is known for this fluorophore and for other fluorophores (see web information from Atto manufacturer (www.atto‐tec.com) and [31, 32]). The absorbance of Agp2 bound fluorophore was estimated to be 0.7 × lower that free fluorophore. This value was estimated based on a test labeling with defined concentrations followed by ultrafiltration to obtain the concentration of free dye.

After 2‐h incubation at 20 °C, free Atto was removed by NAP‐5 columns (GE Healthcare) according to the instructions of the manufacturer. Additional concentration/dilution to further remove residual‐free Atto was performed using centrifugal filter units (Amicon® Ultra − 15, Ultracel – 30 K, Merck Millipore Ltd, Darmstadt, Germany). The final samples were characterized by UV/vis spectroscopy.

### Irradiation, photometry, and FRET measurements

The spectral measurements were performed in a dark room with green save light. In darkness, Agp1 is in the Pr and Agp2 is in the Pfr form. For photoconversion, the samples were irradiated with red (32 μmol·m^−2^·s^−1^, 655 nm) or far‐red (200 μmol·m^−2^·s^−1^, 750 nm) light emitting diodes for 2 min.

UV/vis spectra were recorded with a Jasco V550 photometer at a scan speed of 1000 nm·min^−1^. Fluorescence emission spectra were recorded with a Jasco FP 8300 fluorimeter. The excitation wavelength was 470 nm, slit width of excitation and emission light paths were 2.5 nm, and the scan speed was 200 nm·min^−1^. For FRET, Atto‐495‐labeled Agp2 served as donor, Atto‐565‐labeled Agp1 as acceptor. The concentrations of Agp1 and Agp2 stock solutions were adjusted to A_700 nm_ and A_750 nm_ of 0.2, and equal volumes were mixed. For measurements on single samples, the stock solution was mixed with equal volume of buffer. The absorbance and fluorescence spectra of the Agp1 and Agp2 components in the mixtures were obtained by decomposing the spectra of the mixtures using the respective absorption and fluorescence spectra of the components (before mixing). For decomposition, we used a|e – uv–vis‐ir spectral Software (Søren Preus, http://www.fluortools.com).

To estimate donor quenching (reduced acceptor fluorescence by donor addition), we calculated the energy transfer efficiency *E*_FRET_ according to Formula 1.

(1) $$E_{\text{FRET}}=1-\frac{I_{\mathrm{DA}}A_{D}}{I_{D}A_{\mathrm{DA}}}$$

where *I*_*DA*_ is the integrated fluorescence intensity of the donor emission in the presence of the acceptor, *I*_*D*_ is the integrated fluorescence intensity of the donor emission of the donor alone. *A*_*D*_ is the absorbance of the donor without acceptor at the excitation wavelength (here 470 nm) and *A*_*DA*_ the absorbance of the donor in the presence of the acceptor (in the mixture) at the excitation wavelength. The absorbance values of the fluorophore were calculated from the absorbance spectra after decomposition.

The stimulated emission (donor emission increased by acceptor) was calculated according to Formula 2.

(2) $$E_{\text{FRET}}=\frac{I_{\mathrm{AD}}A_{A}-I_{A}A_{\mathrm{AD}}}{I_{A}A_{\mathrm{DA}}}$$

*A*_*AD*_ is the absorbance of the acceptor in the mixed sample, *I*_*AD*_ is the emission intensity of the acceptor, obtained from the emission spectrum of the mixture by decomposition, *I*_*A*_ is the integrated emission of the acceptor alone. *A*_*A*_ is the absorbance of the acceptor at the emission wavelength, and *A*_*DA*_ the absorbance of the donor in the presence of the acceptor (in the mixture) and *A*_*AD*_ is the absorbance of the acceptor at the excitation wavelength in the mixed sample.

In cases where nonirradiated and irradiated samples were compared, we estimated the fluorescence ratios between 590 nm (acceptor maximum) and 523 nm (donor maximum) and compared these with each other.

### Modeling

Full‐length Agp1 and Agp2 dimers were modeled with alphafold 2 [33], interaction between Agp1 and Agp2 were simulated using cluspro [34], an advanced protein–protein docking program that uses multiple strategies to explore a comprehensive set of possible receptor‐ligand interactions. It uses a Fast Fourier Transform (FFT)‐based calculation of the interaction energy, which incorporates correlation functions for attractive and repulsive van der Waals interactions and electrostatic terms. In the rigid‐body approximation, this approach enables the simultaneous calculation of all translations, resulting in the generation of billions of conformations and a wide range of docking results. cluspro effectively groups similar docking configurations and provides a diverse set of possible binding orientations, ensuring a thorough exploration of potential interaction scenarios between Agp1 and Agp2.

All 40 selected interaction models were used for distance measurements between Agp1 and Agp2 label positions. Every donor fluorophore position in the Agp2 dimer and every acceptor fluorophore position in the Agp1 dimer were determined based on the pdb file, which contains coordinates of all atoms of Agp1 and Agp2 dimers. Using the *x*, *y*, and *z* coordinates of the C_ß_ of the 14 cysteine positions in the Agp2 dimer and the two cysteine positions in the Agp1 dimer, we calculated all relevant donor–acceptor distances. These distances are critical for determining the potential for FRET. The C_ß_ atom is few Å away from the true fluorophore position. We did not try to predict the fluorophore position more precisely. For each of the 28 Agp1‐Agp2 distances (*r*), *E* values were calculated by Formula 3.

(3) $$E_{\text{FRET}}=1/\left(1+{\left(r/R\right)}^{6}\right)$$

with *R* = 50 Å (according to the manufacturer). The total *E*_FRET_ value was calculated by Formulae 4 and 5.

(4) $$E_{1}=\sum E_{1j}$$

(5) $$E_{2}=\sum E_{2j}$$

(*E*_1_ and *E*_2_ represent the labels on the first and the second Agp1 monomer, *j* refers to all positions on either subunit of the Agp2 dimer. *E*_1*j*_ and *E*_2*j*_ describe the *E*_FRET_ between the first monomer of Agp1 and the *j*‐th position on Agp2, and between the second monomer of Agp1 and the *j*‐th position on Agp2, respectively.)

(6) $$E_{\text{total}}=1-\left(1-E_{1}\right)\left(1-E_{2}\right)$$

This formula integrates the values from both Agp1 subunits.

## Results

Since both phytochromes Agp1 and Agp2 jointly regulate *A. fabrum* conjugation and gene transfer [14, 15], it has been suggested that both may also physically interact. Mixing recombinant Agp1 and Agp2 showed that one protein can affect spectral properties or phosphorylation of the other, suggesting that both interact indeed *in vitro*. Also, FRET measurements have been performed with the K517C mutant of Agp1 and wild‐type Agp2. These measurements confirmed the interaction [16]. The attachment of the fluorescent label in the present measurement occurs through Cys residues via the maleimide group of the Atto dye. In this study, the FRET measurements were expanded by using diverse Agp1 Cys mutants to gain spatial information of the interaction. These are characterized by one free cysteine per Agp1 monomer as covalent Atto‐binding site. In earlier studies, these mutants were tested for autophosphosphorylation. In the Pfr state, phosphorylation of Agp1 wild‐type was reduced as compared to Pr [25], and this pattern was reproduced in all mutants with the exception of K554C, where equal phosphorylation was found for both forms. Pr and Pfr spectra were indistinguishable between the mutants. The phosphorylation assays and the spectral properties suggested that the mutations did not affect the overall structure of Agp1 significantly. Agp2 has seven free Cys, besides the chromophore binding Cys13. In most cases, this wild‐type protein was used as the other compound in FRET. The models with label‐binding sites are shown in Fig. 1.

### Labeling of Agp1 and Agp2 and mutants

**Fig. 2:**
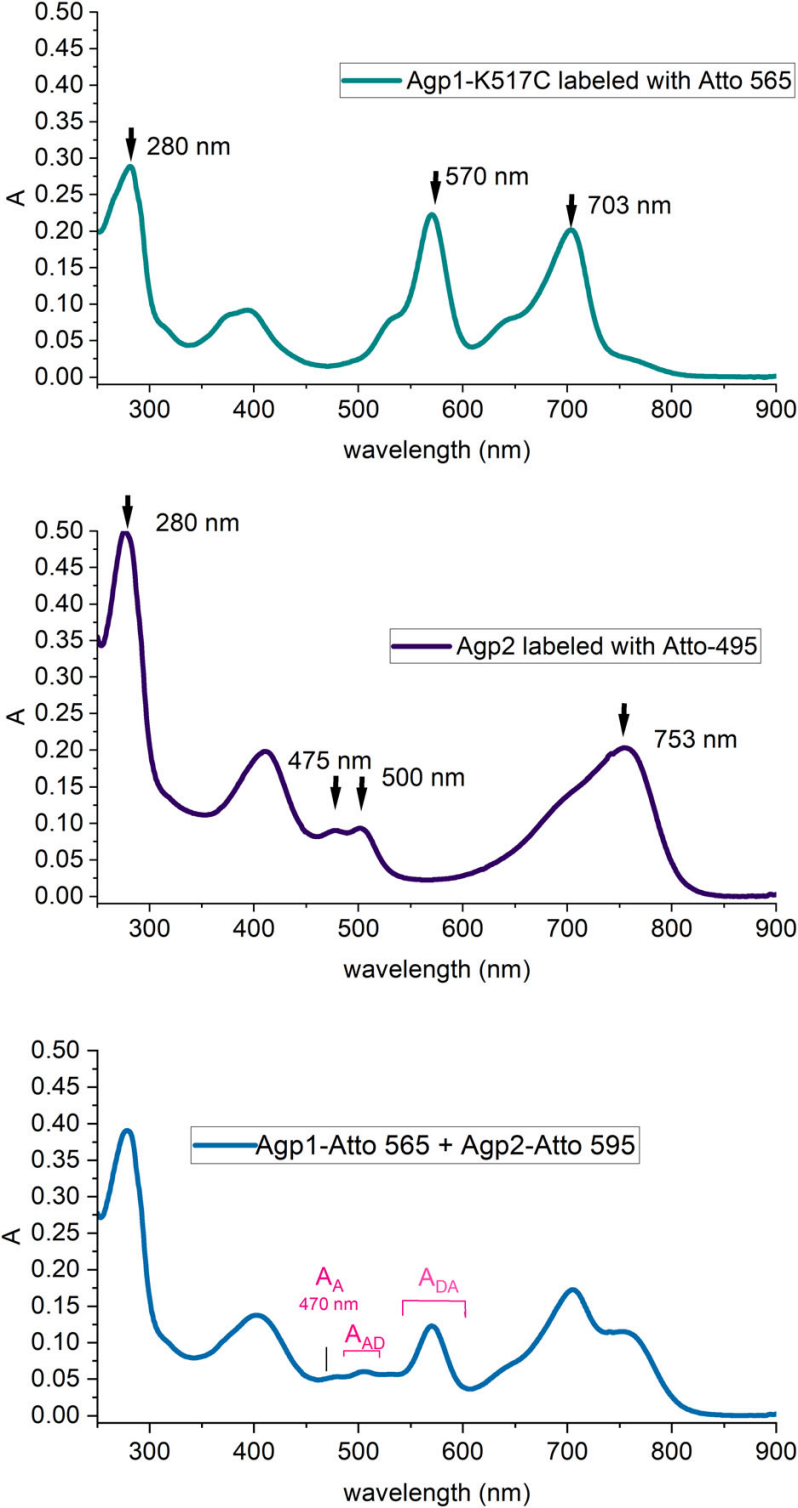
Absorption spectra of Atto labeled Agp1 and Agp2. The positions off relevant maxima are indicated with black arrows and numbers, the *A*_*DA*_ and *A*_*AD*_ ranges are indicated in magenta. These integrated values are obtained after decomposition.

Agp2 variants were always labeled with Atto‐495, these proteins served as donor for the FRET measurements. Agp1 variants, labeled with Atto‐565, served as acceptor. Figure 2 shows absorbance spectra of labeled Agp1‐K517C and labeled Agp2 as examples. From such spectra, the amounts of BV and Atto dye incorporated were estimated. For Agp1, the BV incorporations were between 64% and 78% and Atto‐495 incorporations between 83% and 93% (Table 1). Assembled samples are thus a mixture of a low fraction of apoprotein and holoprotein. According to our experience, apo and holoprotein are biochemically and structurally similar, though they are not identical. The key difference is that photoconversion is not possible for the apoprotein. Autophosphorylation of Agp1 apoprotein is slightly stronger than that of holoprotein‐Pr or Pfr. In interpreting the present data, we assume that the structural differences between the apo‐ and holoprotein forms are negligible for the purposes of this study.

**Table 1:** Estimations for BV incorporation and incorporation of Atto 565 per Agp1 monomers, wild type and mutants proteins, based on absorbance spectra. Besides the chromophore binding Cys20, the mutants have only one Cys that might serve as attachment site for Atto 565. After assembly and after incorporation with twofold molar excess of Atto 565, free molecules were removed by NAP desalting columns. The A_280 nm_ value was taken as measure for the protein concentration, A_700 nm_ was taken as measure for the concentration of the BV chromophore, the A_570 nm_ value as measure for Atto‐565. Extinction coefficients used for the calculations are given in the Materials and methods section.

	S122C	C295	A362C	A362C‐PCM	K517C	K554C	R603C
Molecular ratio 703 nm/280 nm	0.69 ± 0.04	0.79 ± 0.02	0.82 ± 0.01	0.69 ± 0.1	0.77 ± 0.02	0.65 ± 0.07	0.76 ± 0.02
Molecular ratio 570 nm/280 nm	1.1 ± 0.04	0.88 ± 0.01	0.95 ± 0.02	0.90 ± 0.05	0.93 ± 0.02	0.51 ± 0.3	0.83 ± 0.02

For S122C, a mean value of 1.1 of Atto was obtained. This value above 1 suggests that at least one parameter in our calculation might be slightly inaccurate, but it is not expected that this leads to false conclusions.

Agp2 was mainly used as wild‐type protein. We also generated mutants C47S, C249S, C277S, and C353S with the final goal to make mutants with single Cys residues, but the strategy failed as outlined below.

The molar incorporation ratio of the BV chromophore into wild‐type Agp2 was 0.7 (Table 2). The mutants C249S and C277S had a low BV incorporation yield of ca. 0.3 (Table 2). The low BV incorporation suggested that these mutations affect the folding of the chromophore pocket. If even more Cys were mutated, we expected that negative effects could sum up and result in a misfolded Agp2, which cannot be used for interaction studies. We therefore stopped our mutagenesis strategy and continued with wild‐type Agp2. For fluorophore labeling, we used a low Atto/Agp2 ratio in order to have less than one label per protein. The incorporated fluorophore had a dual‐band absorbance with peaks at 475 nm and at 500 nm. A molar fluorophore to protein ratio of 0.16 for Agp2 monomer was calculated based on the 500 nm band and the extinction coefficient of free Atto. Considering a 0.7‐fold reduction in absorbance during incorporation, this corresponds to an Atto/protein ratio of 0.24 for the monomer and 0.48 for the dimer. For subsequent simulation studies, we assumed one label per Agp2 dimer. The incorporation of two labels is unlikely, and a protein with no label does not fall into account. We were not able to determine the exact distribution of fluorophore over the seven Cys residues of Agp2. According to structural models, Cys47, Cys249, and Cys 352 are exposed on the protein surface, while the sulfurs of Cys29, Cys33, Cys248, or Cys647 are 4–6 Å away from the surface and not shielded by other amino acids, that is, directly accessible. Note that Atto‐565 incorporation yields into the different Agp1 mutants were always similar (Table 1). For these reasons, we assume an equal distribution of labels among Agp2 cysteines. This distribution will later be important in the simulation studies. In test cases, deviations thereof are considered.

**Table 2:** Estimations for chromophore incorporation and incorporation of Atto‐495 per protein in Agp2 monomer, wild‐type and mutants, based on absorbance spectra. Besides the chromophore binding Cys13, Agp2 has seven Cys that might serve as attachment site for Atto‐495. After BV assembly and after incorporation with Atto‐495/Agp2 ratio of 1, free molecules were removed by NAP desalting columns. Note that the absorbance spectrum of incorporated Atto‐495 differed from the free fluorophore as the extinction coefficient is 0.7‐fold lower. The presented values are uncorrected. The double band in the spectrum indicates interactions of two or more species. Only the 500 nm band resembles the fluorescent species. The A_280 nm_ value was taken as measure for the protein, A_753 nm_ was taken as measure for BV chromophore, the A_500 nm_ value as measure for Atto‐495. Extinction coefficients used for the calculations are given in the Materials and methods section.

	Agp2	Agp2 PCM	Agp2 HK	Agp2 C47S	Agp2 C249S	Agp2 C277S	Agp2 C353S
Molecular ratio chromophore 753 nm/280 nm	0.70 ± 0.04	0.98 ± 0.01		0.69 ± 0.04	0.36 ± 0.05	0.32 ± 0.02	0.68 ± 0.07
Uncorrected molecular ratio Atto‐protein	0.16 ± 0.01	0.17 ± 0.01	0.52 ± 0.03	0.18 ± 0.01	0.43 ± 0.1	0.39 ± 0.1	0.37 ± 0.05

### FRET measurement

**Fig. 3:**
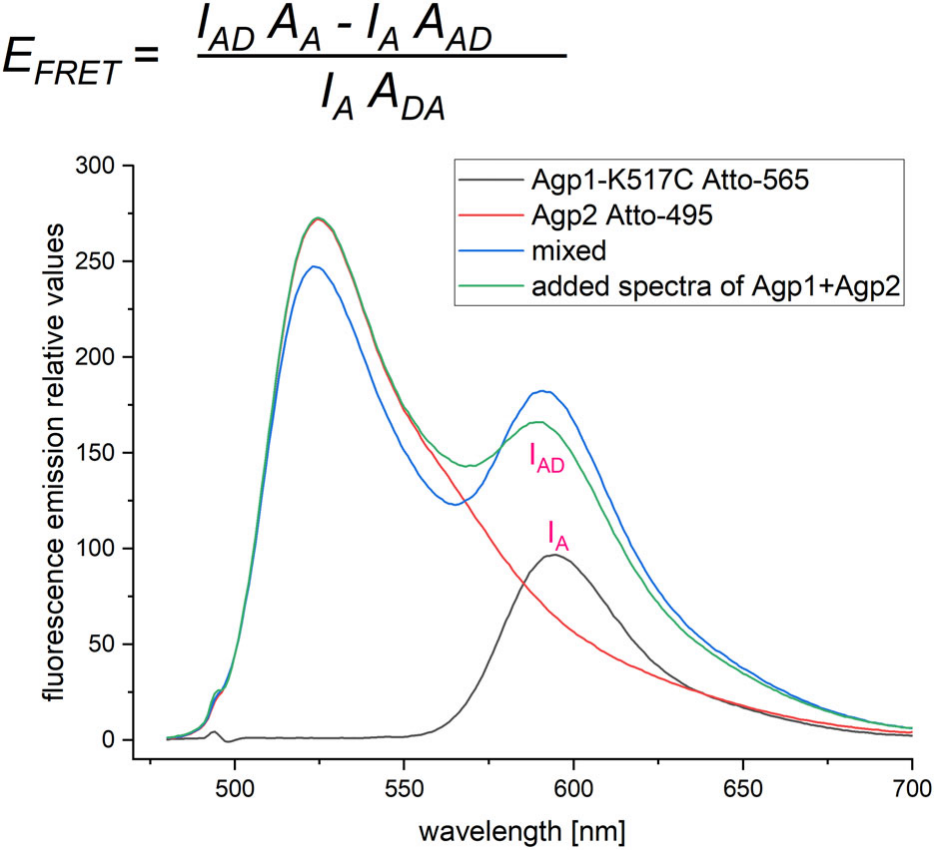
Fluorescence emission spectra of Atto labeled Agp1 and Agp2 as indicated in the annotation of the diagram. The samples were excited at 470 nm. Note that the spectra of the mixed sample (blue) has a higher 595 nm fluorescence than the added spectra (green). The formula for stimulated emission is given above the diagram. The integrated value for *I*_*DA*_ was estimated after decomposition, the integrated value for IA was estimated directly.

In general, UV/vis spectra (Fig. 2) and fluorescence emission spectra of these three samples were measured. For fluorescence emission spectra, the excitation wavelength was always set to 470 nm. Figure 3 shows fluorescence spectra for Atto‐565‐labeled Agp1‐K517C and Atto‐495‐labeled Agp2 and a mixed sample. Despite the low incorporation rate of Atto‐495 into Agp2, a clear fluorescence emission signal with a maximum at 530 nm was always obtained. Since the acceptor also absorbs light at 470 nm, its emission band at 595 nm was still comparably high. In the mixed sample, FRET resulted in an increase of the 595 nm emission band, on top of the emission band of the acceptor. A comparison of the emission spectrum of the mixed sample and the spectrum calculated from the sum of the emission spectra of the donor and acceptor alone showed that FRET occurred (Fig. 3). This pattern was found for all measurements and confirms the interaction of Agp1 and Agp2 [16].

We measured the fluorescence of every labeled Agp1 mutant in combination with labeled Agp2, as well as the specific combinations of Agp2‐PCM with Agp1‐K517C and Agp1‐PCM‐A362C with Agp2. The conversion of the calculated *E*_FRET_ values into distance information is dependent on fluorophore occupancy and several other parameters. The multiple labels per protein dimer makes exact distance calculations almost impossible. However, the *E*_FRET_ values can be used in conjunction with docking studies to provide spatial information, as detailed below.

Donor quenching (donor fluorescence decreased by acceptor) and stimulated emission (acceptor fluorescence increased by donor) values were calculated as given in the Materials and methods section. The relevant parameters for stimulated emission are also indicated in Figs 2 and 3. The donor quenching and stimulated emission approach yielded comparable results for energy transfer, but the errors were larger for the donor quenching results. We therefore present the data of stimulated emission here (Table 3).

**Table 3:** FRET measurements between multilabeled Agp2 and Agp1 labeled at selected positions. *E*_FRET_ gives the estimated FRET transfer efficiency, mean value ± SE of four independent measurements. Equal letters (a, b) indicate significant differences between the respective samples, ** means difference to all other samples was significant. The (1/*E*−1)^(1/6)^ column gives the relative distances. An apparent distance *r* is calculated from this value by multiplication with *R* = 50 Å. The meaning of *r* is discussed in the text.

Donor	Acceptor Agp1…	*E*_FRET_	(1/*E*−1)^(1/6)^	*r* in Å
Agp2	S122C	0.09 ± 0.02 ab	1.47 ± 0.04	73 ± 2
Agp2	A362C	0.10 ± 0.02	1.45 ± 0.04	72 ± 3
Agp2	K517C	0.15 ± 0.04	1.34 ± 0.05	67 ± 3
Agp2	R535C	0.21 ± 0.02 a	1.24 ± 0.03	62 ± 2
Agp2	K554C	0.28 ± 0.06 b	1.17 ± 0.05	58 ± 3
Agp2	R603C	0.20 ± 0.03	1.26 ± 0.03	63 ± 2
Agp2	A362C PCM	0.00 ± 0.03 **	—	—
Agp2‐PCM	K517C	0.14 ± 0.03	1.35 ± 0.05	67 ± 3

The mean values of FRET efficiencies of the different Agp1 mutants with Agp2 (Table 4) increased from S122C (*E*_FRET_ = 0.09) over A362C, K517C, R535C, to K554C (*E*_FRET_ = 0.28). The mutant with a Cys at the most C‐terminal position, R603C, had again a lower value than K554C. The biggest difference was between K554C and S122C. Differences between S122C and R535C and between S122 and K554C were significant (Table 3). When the labeled PCM of Agp2 (protein without histidine kinase/response regulator) and labeled Agp1‐K517C were mixed, the FRET signal was almost the same as with full‐length labeled Agp2 as the donor (Table 3). No FRET signal was obtained with labeled Agp1‐PCM‐A362C as acceptor and labeled full‐length Agp2 as donor (Table 3). The differences between Agp1‐PCM and all other Agp1 variants including A362C were significant. The PCM of Agp2 must be involved in the interaction of Agp1 and Agp2, whereas the PCM of Agp1 must not be involved in the interaction. In these measurements, FRET occurs between multiple pairs of fluorophores, as outlined above. Considering the low Atto incorporation into Agp2, the donor had one label per monomer at one of 14 labeled Cys positions in each dimer or less. Protein without label would not contribute to the fluorescence measurement, and the probability for two labels per dimer is low. For Agp1, we assumed an incorporation rate of 2 labels per dimer. One FRET measurement is the result of a mix of labels on seven different Agp2 positions and of two labels on the respective Agp1 mutant. Despite the difficulty to calculate distances, we can nevertheless speak of an apparent distances (*r*), which will allow for further conclusions about the interaction between Agp1 and Agp2. These values were between 58 Å for Ser 122 and 75 Å for Lys 554 (Table 3). When Agp1 and Agp2 dimer models were arranged manually in a parallel or antiparallel way, distances between donor and acceptor labels were between 30 and 70 Å and in the docking models discussed below, distances were between 35 and 130 Å. The apparent r values of the FRET measurements are thus in an appropriate range.

**Table 4:** FRET measurements with dark and irradiated samples, Agp1‐K5117C and Agp2. In the first column, the irradiation for Agp1 and Agp2 is given; d, no irradiation, r, irradiated with red light that predominately converts Pr to Pfr, fr, irradiated with far‐red light that predominately converts Pfr to Pr. Note that Agp1 and Agp2 have a Pr and Pfr dark state, respectively. The second column gives the F_590 nm_/F_523 nm_ ratio as relative value for FRET. *t*‐Test significance of differences is given in the third column. All groups of measurements were tested against each other. If two samples are significantly different, the same letter is given in the last row (error probability < 0.05).

	F_590 nm_/F_523 nm_	Significance
Agp1 d, Agp2 d	0.83 ± 0.02	a, b
Agp1 fr, Agp2 fr	0.89 ± 0.02	a
Agp1 r, Agp2 r	0.87 ± 0.03	
Agp1 r, Agp2 fr	0.83 ± 0.03	c
Agp1 r, Agp2 d	0.90 ± 0.04	d
Agp1 d, Agp2 fr	0.75 ± 0.04	a, b, c, d

### FRET measurements using irradiated phytochromes

The previous FRET measurements were made with Agp1 and Agp2 in the nonirradiated states, which are Pr and Pfr, respectively. There are several examples of light‐dependent interactions of phytochromes with other proteins [17]. We therefore tested for the impact of light on the FRET measurements.

Photoconversion was induced either by irradiating a single protein before mixing or by irradiating both proteins after mixing. For a simple data evaluation, the fluorescence ratio F_595 nm_/F_523 nm_ of the mixed samples was calculated. With increasing FRET, the F_595 nm_ value increases and the F_523 nm_ value decreases and the ratio increases. These ratios give relative values that allow also to detect small differences.

The measurements were performed with Agp1 K517C and Agp2. The values deviated from each other up to 10% (Table 4). The highest fluorescence ratios were obtained for both proteins irradiated with far‐red (Agp1 Pr and Agp2 Pr) and with Agp1 irradiated with red (Agp1 Pfr Agp2 Pfr). The lowest value was obtained for Agp2 irradiated with far‐red (Agp1 Pr and Agp2 Pr). The small Pr/Pfr differences suggest that the interaction between Agp1 and Agp2 takes place in either form. However, the distances between labels of Agp1 and Agp2 can change upon photoconversion, though the mechanism remains unclear.

### Modeling the interaction between Agp1 and Agp2, docking models

In order to transfer FRET information into the spatial organization of Agp1 and Agp2, we generated multiple interaction models, compared their theoretical *E*_FRET_ values with experimental data, and selected the most likely models. First, we generated Agp1 and Agp2 dimer models using alphafold 2 (Fig. 1). We assumed that these dimer models were reliable for the purpose of the present project. A homodimer structure with a parallel head to head arrangement of the monomers corresponds to the general picture of the overall dimer arrangement of bacterial phytochromes [6, 35]. Both Agp1 and Agp2 full‐length proteins behaved as dimers when subjected to SEC [18, 19] with the histidine kinase as a strong dimerization site [19]. The crystal structures of the PCM modules of Agp1 (PDB code: 5HSQ) and Agp2 (PDB code: 6G1Y) are known. Their monomers fit with an RMSD of about 0.6–0.7 Å. For histidine kinases of Agp1 and Agp2, no structure is available yet. The known histidine kinase crystal structures of other proteins (monomer subunits) match with an RMSD of 1.5 to 4 Å and the *Thermotoga maritima* histidine kinase (PDB Code: 4KP4) aligned with an RMSD of 4 Å with the monomer of the Agp1 alphafold model. Cryo‐EM structures of related bacterial phytochromes were recently determined [9, 36]. These showed the same parallel arrangement of monomers. The lower two panels of Fig. 1 show aligned structures of the Agp1 alphafold model and the Cryo EM structures PDB ID: 8UPK and PDB ID: 9EUT. The orientation of histidine kinase in the 9EUT model is rotated along the longitudinal axis against the two other structures by ca 90°, whereas 8UPK matches well also in the histidine kinase region with the alphafold model of Agp1. We assume that for the present docking analyses and distance calculations, the possible different arrangements of histidine kinases are irrelevant.

The entire phytochrome protein undergoes conformational changes during photoconversion, though these changes are not completely understood [19, 37]. However, the FRET measurements presented here were only slightly different between combinations of Pr and Pfr. Therefore, it is likely not critical for the present approach, which form is being considered.

Based on the Agp1 and Agp2 dimer models, 40 interaction models were generated by protein docking using the cluspro 2.0 server [34, 38, 39], considering Agp2 as receptor and Agp1 as ligand. To this end, 10 best poses from each of the balanced (model 000.00‐09), electrostatic‐favored (model 002.00‐09), hydrophobic‐favored (model 004.00‐09), and van der Waals‐electrostatic‐favored (model 006.00‐09) models were selected. The 40 docking models should provide all relevant combinations of Agp1 and Agp2 arrangements, since the selection set already contains highly similar arrangements and newly generated cluspro models were always similar to one of the previously generated models. The surface presentation of the models is shown in the Fig. S1.

For each interaction model, expected *E*_FRET_ values were estimated. The final *E*_FRET_ value for each model was calculated from the 28 single *E* values. The calculation assumes that all Agp2 molecules have one fluorophore in a dimer and Agp1 has two fluorophores in the dimer. This means that for the total *E*_FRET_ value for 14 Agp2 label positions, the mean value must be calculated for each Agp1 monomer separately (Formulae 4 and 5 in the Materials and methods section). To obtain the final value for both Agp1 labels, the two *E*_FRET_ values (from Formulae 4 and 5) were used to calculate the product of 1 minus the respective *E*_FRET_ values (Formula 6). The calculated *E*_FRET_ values are presented in Table 5. We also calculated the differences between the experimental data and model E_FRET_ values and the sum of the squares of the differences (Table 5, column 7). In Table 5, it is also given whether the interaction of the model is based on PCM or histidine kinase of Agp1 or Agp2 (last columns). By comparing the data from the docking models with the measured data (Table 5, top line), we tried to find the most realistic model(s) of the docking analysis.

**Table 5:** Computer models for interaction of Agp1 and Agp2. *E*_FRET_ value estimations for each model. The header row (in Columns 2–7) indicates the label position of Agp1, the data in these columns are the estimated *E*_FRET_ values. The right four columns show whether interaction between HK/PCM is expected based on the 3D models. The first column indicates the name of the model, the first row below the header row shows the data for experimentally determined FRET. cluspro models are denominated with ‘model …’, the alphafold model with ‘ranked0’. Sum of DE^2^ stands for the sum of distance squares between measured *E*_FRET_ value and *E*_FRET_ value from the model.

Agp1 amino acid	122	362	517	535	554	603	Sum of DE^2^	Agp1 PCM	Agp1 HK	Agp1 PCM	Agp1 HK
Experimental *E*_FRET_	0.090	0.100	0.150	0.210	0.280	0.200		No	Yes	Yes	No
model.000.00	0.404	0.525	0.211	0.079	0.048	0.057	8.53	Yes	No	Yes	No
model.000.01	0.442	0.467	0.189	0.053	0.025	0.039	9.86	Yes	No	Yes	No
model.000.02	0.428	0.456	0.156	0.045	0.023	0.039	10.37	Yes	No	Yes	No
model.000.03	0.400	0.494	0.182	0.068	0.040	0.047	9.10	Yes	No	Yes	No
model.000.04	0.005	0.091	0.305	0.392	0.350	0.337	0.65	No	Yes	Yes	Yes
model.000.05	0.004	0.075	0.250	0.363	0.338	0.325	0.61	No	Yes	No	Yes
model.000.06	0.005	0.077	0.251	0.358	0.341	0.287	0.57	No	Yes	No	Yes
model.000.07	0.034	0.188	0.134	0.051	0.021	0.079	5.55	No	Yes	Yes	No
model.000.08	0.005	0.128	0.308	0.404	0.376	0.391	0.55	No	Yes	No	Yes
model.000.09	0.004	0.073	0.345	0.600	0.683	0.529	0.67	No	Yes	Yes	No
model.002.00	0.005	0.077	0.251	0.358	0.341	0.287	0.57	No	Yes	No	Yes
model.002.01	0.005	0.091	0.305	0.392	0.350	0.337	0.65	No	Yes	No	Yes
model.002.02	0.004	0.075	0.250	0.363	0.338	0.325	0.61	No	Yes	No	Yes
model.002.03	0.003	0.064	0.297	0.592	0.741	0.559	0.78	No	Yes	Yes	No
model.002.04	0.004	0.073	0.345	0.600	0.683	0.529	0.67	No	Yes	Yes	No
model.002.05	0.004	0.072	0.315	0.541	0.672	0.519	0.67	No	Yes	Yes	No
model.002.06	0.346	0.214	0.062	0.022	0.013	0.012	12.98	Yes	No	Yes	No
model.002.07	0.005	0.128	0.308	0.404	0.376	0.391	0.55	No	Yes	No	Yes
model.002.08	0.357	0.213	0.067	0.027	0.016	0.013	12.58	Yes	No	Yes	No
model.002.09	0.343	0.108	0.049	0.054	0.045	0.018	11.19	Yes	No	Yes	No
model.004.00	0.138	0.328	0.135	0.073	0.039	0.053	7.04	Yes	No	Yes	No
model.004.01	0.186	0.366	0.124	0.034	0.016	0.044	9.92	Yes	No	Yes	No
model.004.02	0.131	0.221	0.142	0.075	0.034	0.044	5.42	Yes	No	Yes	No
model.004.03	0.005	0.113	0.183	0.183	0.151	0.271	1.17	No	Yes	Yes	No
model.004.04	0.019	0.294	0.295	0.311	0.309	0.377	0.81	No	Yes	Yes	No
model.004.05	0.157	0.319	0.091	0.026	0.013	0.036	10.53	Yes	No	Yes	No
model.004.06	0.027	0.348	0.352	0.315	0.294	0.382	1.12	No	Yes	Yes	No
model.004.07	0.005	0.104	0.188	0.184	0.133	0.249	1.27	No	Yes	Yes	No
model.004.08	0.016	0.111	0.098	0.072	0.050	0.163	2.50	Yes	Yes	Yes	Yes
model.004.09	0.014	0.108	0.104	0.079	0.061	0.206	2.61	Yes	Yes	Yes	Yes
model.006.00	0.037	0.400	0.510	0.399	0.304	0.348	1.52	No	Yes	Yes	No
model.006.01	0.036	0.467	0.491	0.384	0.304	0.480	1.60	No	Yes	Yes	No
model.006.02	0.029	0.369	0.534	0.427	0.323	0.472	1.33	No	Yes	Yes	No
model.006.03	0.054	0.349	0.213	0.165	0.149	0.206	2.55	No	Yes	Yes	No
model.006.04	0.055	0.221	0.288	0.234	0.137	0.436	2.03	No	Yes	Yes	No
model.006.05	0.097	0.530	0.344	0.150	0.057	0.217	5.57	Yes	No	Yes	No
model.006.06	0.009	0.173	0.427	0.465	0.396	0.457	0.73	Yes	No	Yes	No
model.006.07	0.034	0.437	0.492	0.396	0.273	0.450	1.67	No	Yes	Yes	No
model.006.08	0.090	0.452	0.318	0.144	0.058	0.211	4.96	Yes	No	Yes	No
model.006.09	0.071	0.514	0.264	0.160	0.109	0.249	4.55	No	Yes	Yes	No
Manual parallel	0.307	0.250	0.191	0.148	0.114	0.101	3.49				
Manual vertical	0.052	0.431	0.316	0.161	0.091	0.301	3.92				
Manual antiparallel	0.087	0.181	0.379	0.400	0.288	0.403	0.72				
alphafold ranked0	0.400	0.591	0.274	0.166	0.037	0.024	8.08				

A direct comparison between measured *E*_FRET_ and docking data is not necessarily a reliable selection criterium, because the measured *E*_FRET_ depends on the labeling efficiencies of Agp2. Therefore, we chose relaxed selection criteria that would be independent on labeling efficiency. Here, rather the differences within a model are considered for selection.

The Selection Criterion 1 was based on the internal difference between Agp1 Positions 122 and 554. These had the lowest and highest *E*_FRET_ values in the measurements, respectively, and the difference was significant (Table 3). Selection Criterion 2 addresses the *E*_FRET_ value at Position 362. This was significantly lower than that at Position 554 in the measurements. The Selection Criterion 3 is related to the PCM data. According to the FRET measurements, Agp1 should interact with Agp2 via its histidine kinase, whereas Agp2 should interact with Agp1 via its PCM (Table 3).

For training purposes, we started with three Agp1/Agp2 interaction models that were manually generated, head to head (parallel), head to tail (antiparallel), and vertical. These structures are presented in Fig. S1. For the antiparallel orientation, but not for the other two, Selection Criteria 1 (122 vs. 554), 2 (362 vs. 554), and 3 (PCM) were fulfilled (Table 5).

**Fig. 4:**
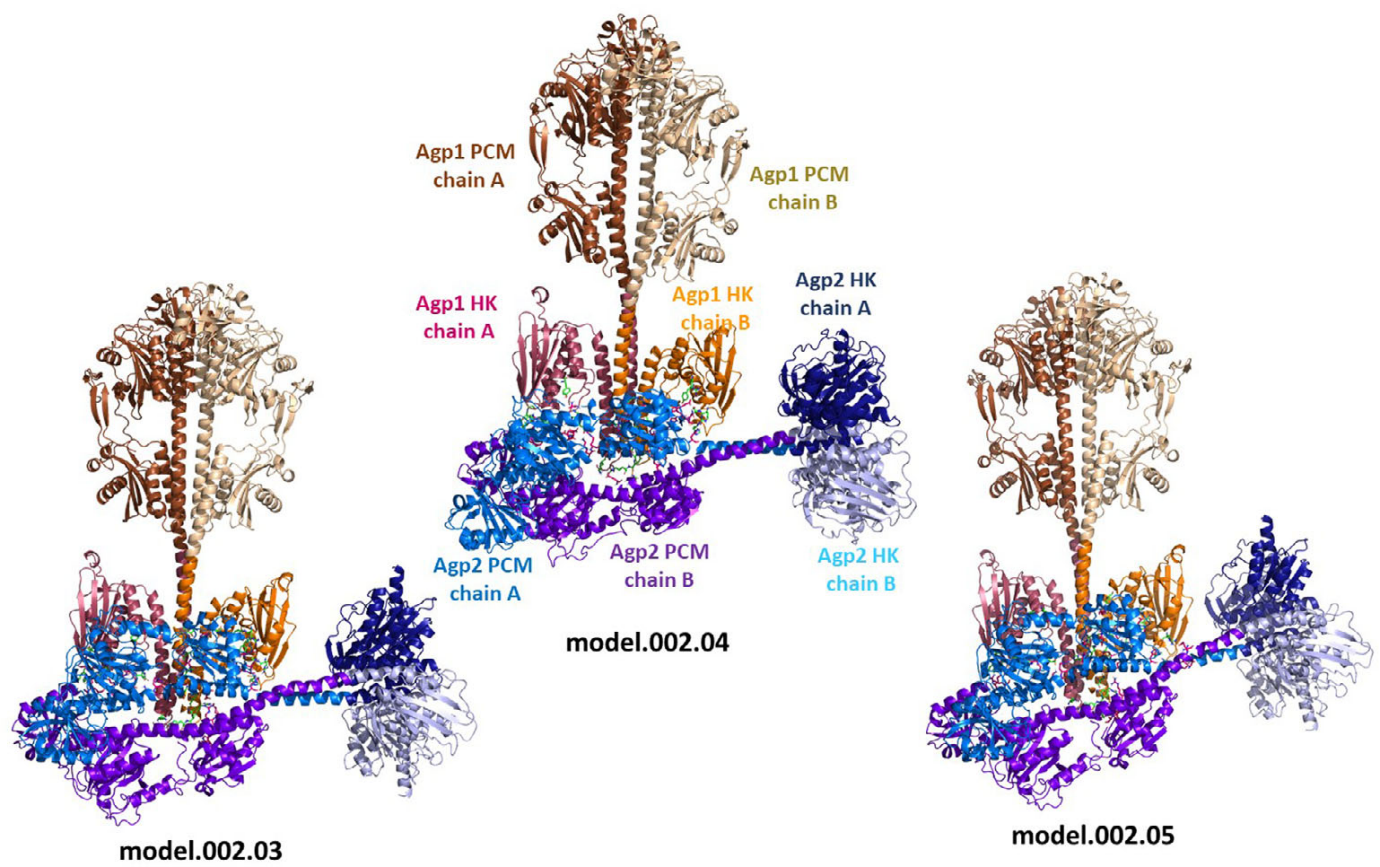
Docking interaction models of Agp1 Agp2 dimers predicted by the cluspro web server. Out of a total of 40 models, three models fit the experimental data, model 002.03, model 002.04 and model 002.05. The color code distinguishes Agp1 and Agp2, PCM and histidine kinases (HK) and chains A and B of the homodimers.

With Selection Criterion 1 (122 vs. 554), 20 of the 40 docking models were excluded (Table 5). The Selection Criterion 2 (362 vs. 554) excluded an additional six models. (Table 5). With Selection Criterion 3 (PCM or histidine kinase), the selection was further narrowed to four models: model 000.09 model 002.03, model 002.04, and model 002.05 (Table 5). These models are highly similar to one another, with models 000.09 and 002.04 being nearly identical. Therefore, model 000.09 was not further considered. Models 002.03, 002.04, and 002.05 are shown in Fig. 4, in addition to the Fig. S1. Four *E*_FRET_ values from these simulations were consistently higher than the experimental values while two were consistently lower.

In these calculations, we assumed an equal distribution of fluorophores among the different positions in Agp2. However, the simulations allow also variable distributions. We tested for model 002.04 how a range of label distributions, varying by a 40‐fold difference, would affect the outcome. The higher *E*_FRET_ value observed at Position 544 compared with Position 122 was robust; no distribution model was able to reverse this pattern. The *E*_FRET_ value at position 362 was consistently low in most cases, often around half that of position 544. An increase in *E*_FRET_ values from the N terminus of Agp1 to Position 544 was also obtained, although at times, Positions 517 and 535 higher values higher than at Position 544. Nonselected models were also tested under variable Agp1 label distributions. The observed difference between high and low values at Positions 122 and 554 (now opposite to the selected models) was robust and could not be reversed. This suggests that the difference between Agp1 at Positions 122 and 554 serves as a robust selection feature for model validation, supporting our conclusion that the selected models are the most likely ones.

**Fig. 5:**
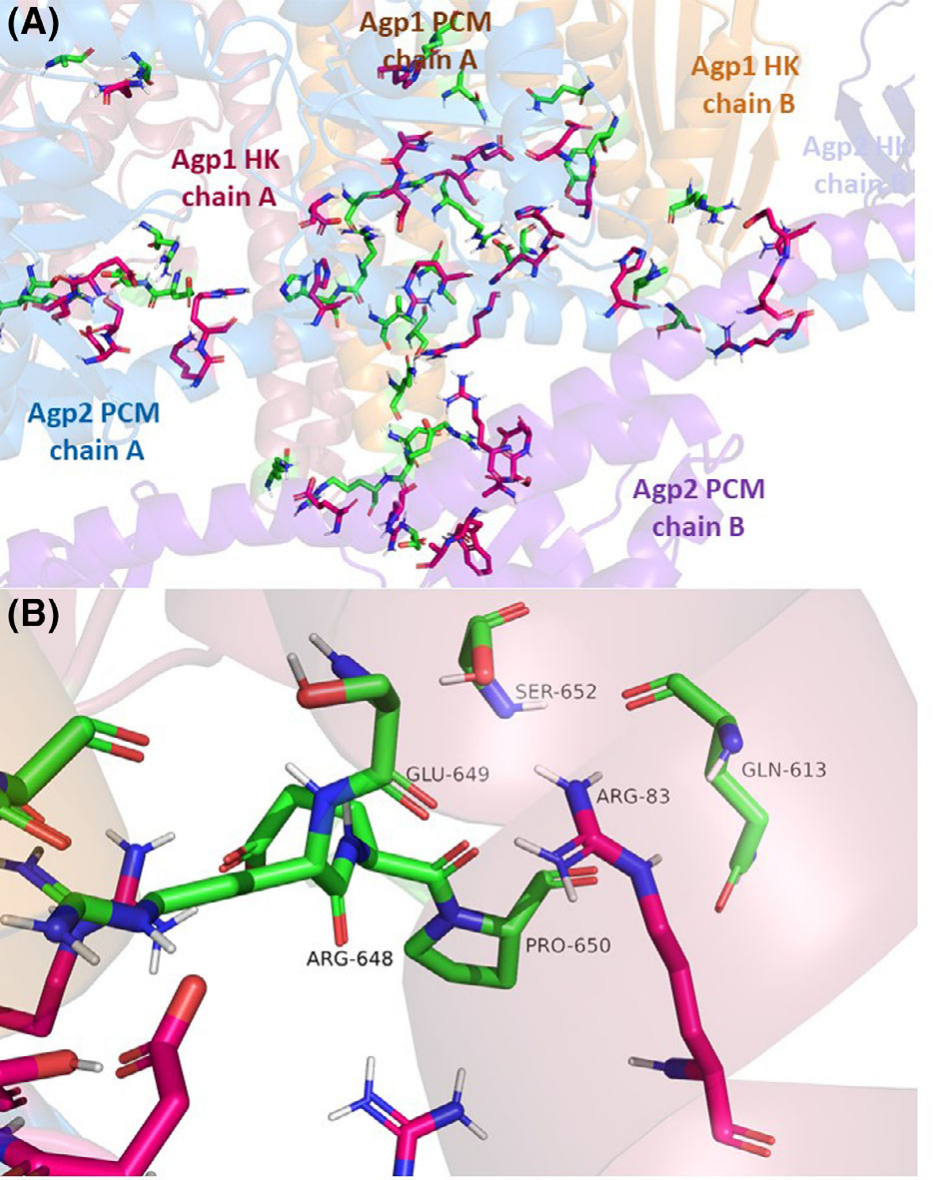
A Overview of interacting amino acids of model002.05. The protein is drawn in transparent cartoon with the same color code as in Fig. 4. Interacting amino acids are drawn in sticks mode with carbon atoms in magenta for Agp2 and in green for Agp1. B Arg83 of Agp2 which interacts with five amino acids of Agp1. The relevant amino acids are drawn in stick mode, color code as in A.

The interaction surfaces of the three models were further analyzed by examining pairs of amino acids in Agp1 and Agp2 that are within 3 Å of each other. Since the modeling program does not account for induced fit adaptations, actual side chain orientations may differ in a real biological context. Depending on the model, 44 to 52 such amino acid pairs were identified. The selection of amino acid pairs was highly similar across models, consistent with the quasi‐identical overall structure. The selected amino acids for these models, sorted by their positions in Agp1, are presented in Table 6. In Figs 4 and 5, interacting amino acids are displayed in stick mode, with distinct colors differentiating Agp1 from Agp2. Our focus is on the pairs common to all three models.

**Table 6:** Pairs of amino acids in the models 002.03, 002.04, and 002.05. Name, number (#), and chain of the relevant amino acid are given in the first, second, and third column, the left three columns for Agp1, followed by Agp2 amino acids, followed by the distance between both amino acids. Amino acids that belong to the same region/group (see text) have a background with the same color. Amino acids that do not belong to a group have a white background. Bold letters and numbers indicate two neighbouring amino acids (position # ‐+ 1)

002.03							002.04							002.05						
Agp1	#	Ch	Agp2	#	Ch	Dist	Agp1	#	Ch	Agp2	#	Ch	Dist	Agp1	#	Ch	Agp2	#	Ch	Dist
ARG	531	B	ASP	346	A	1.74	TYR	524	B	HIS	367	A	2.09	ARG	531	B	**ASP**	**342**	**A**	**1.78**
**ARG**	**535**	**B**	HIS	318	A	1.76	ARG	531	B	**GLN**	**345**	**A**	**1.89**	ARG	531	B	**SER**	**343**	**A**	**1.84**
**ARG**	**535**	**B**	ASP	349	A	1.78	ARG	531	B	**ASP**	**346**	**A**	**2.64**	ARG	531	B	**GLN**	**345**	**A**	**2.67**
**HIS**	**536**	**B**	HIS	318	A	2.5	ARG	535	B	HIS	318	A	2.73	ARG	531	B	**ASP**	**346**	**A**	**1.85**
VAL	538	B	**ARG**	**325**	**A**	**2.75**	ARG	535	B	**ARG**	**325**	**A**	**2.6**	**ARG**	**535**	**B**	HIS	318	A	2.8
GLN	542	B	HIS	320	A	1.97	ARG	535	B	**ASP**	**346**	**A**	**1.85**	**ARG**	**535**	**B**	**ASP**	**346**	**A**	**1.81**
GLN	542	B	**ASP**	**324**	**A**	**2.67**	ARG	535	B	ASP	349	A	1.91	**ARG**	**535**	**B**	ASP	349	A	1.84
**ARG**	**545**	**B**	ALA	386	B	1.77	GLY	539	B	**ARG**	**325**	**A**	**2.31**	**HIS**	**536**	**B**	HIS	318	A	2.55
**ARG**	**545**	**B**	GLU	388	B	1.82	GLN	542	B	ALA	321	A	2.14	VAL	538	B	ARG	328	A	2.42
**GLU**	**546**	**B**	ARG	390	B	1.94	GLN	542	B	**ASP**	**324**	**A**	**2.13**	GLN	542	B	ARG	325	A	3
**GLU**	**546**	**B**	ARG	482	B	1.7	GLN	542	B	ARG	325	A	1.85	GLN	542	B	ARG	328	A	2.69
**ARG**	**547**	**B**	GLU	479	B	1.85	**ARG**	**545**	**B**	GLU	388	B	1.78	GLN	542	B	ARG	390	B	2.68
ASP	549	B	HIS	395	B	2.78	**GLU**	**546**	**B**	ARG	390	B	1.79	**ARG**	**545**	**B**	**ALA**	**386**	**B**	**2.17**
ASP	549	B	ARG	482	B	1.75	**GLU**	**546**	**B**	ARG	482	B	2.01	**ARG**	**545**	**B**	**SER**	**387**	**B**	**2.45**
LYS	554	A	GLU	479	B	1.75	**ARG**	**547**	**B**	GLU	479	B	2.02	**ARG**	**545**	**B**	**GLU**	**388**	**B**	**1.85**
**GLU**	**564**	**B**	ARG	328	A	1.85	ASP	549	B	TRP	392	B	1.96	**GLU**	**546**	**B**	ARG	390	B	1.97
**ARG**	**603**	**B**	**ASN**	**335**	**A**	**1.84**	ASP	549	B	ARG	482	B	1.86	**GLU**	**546**	**B**	ARG	482	B	1.87
**ARG**	**603**	**B**	HIS	499	A	1.93	LYS	554	A	GLU	479	B	1.78	**ARG**	**547**	**B**	GLU	479	B	1.99
**SER**	**604**	**B**	**HIS**	**332**	**A**	**2.94**	ASP	574	B	**GLN**	**345**	**A**	**1.99**	ASP	549	B	**TRP**	**392**	**B**	**2.94**
**SER**	**604**	**B**	**ALA**	**334**	**A**	**1.96**	ASN	578	B	ASP	342	A	2.68	ASP	549	B	**ALA**	**393**	**B**	**2.12**
**SER**	**604**	**B**	GLU	338	A	1.97	**ARG**	**603**	**B**	HIS	499	A	1.86	ASP	549	B	ARG	482	B	1.78
HIS	607	B	HIS	499	A	2.13	**SER**	**604**	**B**	**ALA**	**334**	**A**	**2.25**	LYS	554	A	GLU	479	B	1.81
**ARG**	**612**	**A**	ARG	83	B	2.82	**SER**	**604**	**B**	**ASN**	**335**	**A**	**2.71**	SER	563	B	ARG	328	A	2.1
**GLN**	**613**	**A**	ARG	83	B	1.77	**SER**	**604**	**B**	GLU	338	A	2.06	LEU	567	B	ARG	325	A	2.68
**GLN**	**613**	**A**	ARG	107	B	1.7	HIS	607	B	**ALA**	**334**	**A**	**2.97**	ARG	571	B	**HIS**	**332**	**A**	**2.91**
ARG	632	B	GLU	338	A	1.74	HIS	607	B	HIS	499	A	2	ARG	571	B	**HIS**	**333**	**A**	**2.01**
TYR	636	B	**HIS**	**333**	**A**	**1.97**	GLN	613	A	ARG	83	B	1.94	ASN	578	B	**ASP**	**342**	**A**	**2**
**SER**	**647**	**A**	ARG	83	B	2.22	**ARG**	**632**	**B**	GLU	338	A	1.9	GLN	581	B	HIS	367	A	2.24
**ARG**	**648**	**A**	**ASP**	**140**	**A**	**1.79**	**GLN**	**633**	**B**	ASP	342	A	2.32	ARG	603	B	ARG	507	A	2.8
**ARG**	**648**	**A**	**SER**	**144**	**A**	**2.22**	TYR	636	B	**HIS**	**333**	**A**	**2.01**	ARG	603	B	GLU	508	B	1.79
**GLU**	**649**	**A**	ARG	83	B	2.62	**SER**	**647**	**A**	ARG	83	B	2.48	HIS	607	B	HIS	499	A	2.97
**GLU**	**649**	**A**	ARG	135	A	2.96	**ARG**	**648**	**A**	GLU	138	A	1.8	SER	610	B	ARG	509	A	1.81
**PRO**	**650**	**A**	ARG	83	B	2.1	**ARG**	**648**	**A**	**ASP**	**140**	**A**	**1.84**	GLN	613	A	ARG	83	B	2.22
**PRO**	**650**	**A**	ARG	135	A	2.63	**ARG**	**648**	**A**	**SER**	**144**	**A**	**2.62**	GLN	633	B	GLU	338	A	2.05
SER	652	A	ARG	83	B	1.69	**ARG**	**648**	**A**	ARG	148	A	2.43	TYR	636	B	**HIS**	**333**	**A**	**1.9**
TYR	680	A	ASN	439	A	2.25	**GLU**	**649**	**A**	ARG	83	B	2.66	TYR	636	B	ASN	335	A	2.96
ARG	693	A	**SER**	**141**	**A**	**2.75**	**GLU**	**649**	**A**	ARG	135	A	1.82	**SER**	**647**	**A**	ARG	83	B	2.58
ARG	693	A	**GLU**	**143**	**A**	**1.78**	**GLU**	**649**	**A**	ARG	151	A	1.83	**ARG**	**648**	**A**	**ASP**	**140**	**A**	**1.87**
**GLU**	**695**	**A**	LYS	310	A	1.79	**PRO**	**650**	**A**	ARG	83	B	2.06	**ARG**	**648**	**A**	**SER**	**141**	**A**	**2.31**
**GLU**	**695**	**A**	ARG	311	A	1.78	SER	652	A	ARG	83	B	1.76	**ARG**	**648**	**A**	**SER**	**144**	**A**	**2.83**
**ASP**	**696**	**A**	**ASP**	**140**	**A**	**2.95**	GLY	673	A	ARG	151	A	2.86	**GLU**	**649**	**A**	ARG	83	B	2.27
**ASP**	**696**	**A**	**SER**	**141**	**A**	**2.02**	ALA	679	A	ASN	439	A	1.91	**GLU**	**649**	**A**	ARG	151	A	1.84
							TYR	671	A	ASN	439	A	2.41	**PRO**	**650**	**A**	ARG	83	B	2.18
							ARG	684	A	**GLU**	**143**	**A**	**1.86**	**PRO**	**650**	**A**	ARG	135	A	2.88
							ARG	684	A	**ARG**	**311**	**A**	**2.88**	SER	652	A	ARG	83	B	2.56
							**GLU**	**686**	**A**	**LYS**	**310**	**A**	**1.84**	ALA	670	A	ASN	439	A	2.98
							**GLU**	**686**	**A**	**ARG**	**311**	**A**	**1.99**	LYS	674	A	ASN	439	A	2.07
							**ASP**	**687**	**A**	**SER**	**141**	**A**	**1.93**	ARG	684	A	**GLU**	**143**	**A**	**1.8**
							**ASP**	**687**	**A**	**SER**	**144**	**A**	**2.04**	**GLU**	**686**	**A**	**LYS**	**310**	**A**	**1.75**
							GLU	717	A	LYS	154	A	1.73	**GLU**	**686**	**A**	**ARG**	**311**	**A**	**2**
														**ASP**	**687**	**A**	**SER**	**141**	**A**	**1.94**
														GLU	689	B	**HIS**	**332**	**A**	**2.14**
														GLY	716	A	ARG	151	A	1.9

The identified amino acids of Agp1 largely group into three main regions. The first group, on Agp1 (Positions 524–549, marked in green in Table 6), is located on the long helix connecting the PHY domain and the histidine kinase of chain A, surrounding the substrate histidine at Position 528. Group 2 on Agp1 (blue fields in Table 6) is in the ATPase region of the histidine kinase, spanning residues 563–610 on chain A and encompassing two short helices. Group 3 (light red fields in Table 6) spans Positions 612–713 on chain B and includes two short helices and two β‐sheets in the ATPase region. Groups 2 and 3 are considered distinct groups as they occur on separate chains.

In Agp2, four groups can be distinguished. Group 1 (green fields in Table 6, Positions 135–154) lies in a helix–loop–helix region of the PAS domain on chain A. Group 2 (orange fields in Table 6, Positions 310–349) spans the C‐terminal end of the long GAF‐PHY connecting helix on chain A; within this group, four consecutive amino acids (332–335) cover the helix end and connecting loop. Group 3 (yellow fields in Table 6, positions 386–395) lies on a helix–loop–sheet region of the PHY domain on chain B. Group 4 (red fields in Table 6, Positions 479–509) lies on the long helix connecting the PHY domain to the histidine kinase.

Each group of Agp1 interacts with multiple groups in the partner protein (Table 6). For Agp2, the pattern is different: Group 1 amino acids interact exclusively with Group 1 of Agp1, while Group 3 amino acids of Agp2 interact exclusively with Group 3 of Agp1.

The grouped amino acids likely represent nonspecific interactions, suggesting that the surfaces of Agp1 and Agp2 align somewhat incidentally rather than as a result of coevolution to strengthen these interactions. In contrast, individual amino acids that are not part of any group are more likely to have evolved specifically for interaction. Notably, one such amino acid on Agp1 and two on Agp2 stand out.

**Fig. 6:**
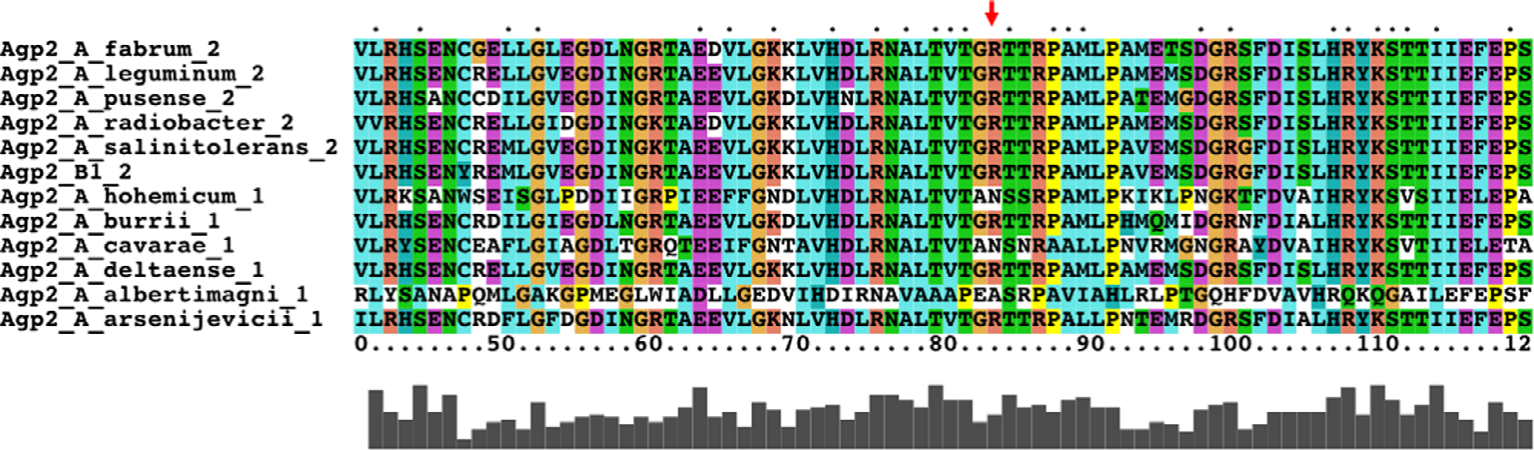
Alignment of Agp2 homologs from 12 species. The species names are given in each header. The number 1 or 2 stands for the number of phytochromes in this species. 1 means only Agp2 homolog, 2 means Agp1 and Agp2 homolog.

In Agp1, the unique Lys554 (white field in Table 6) interacts with Glu479 of Agp2, Group 4 (Table 6). One unique amino acid on Agp2, Asn439, interacts with Ala670 or Tyr671 or Tyr 680 of Agp1 Group 3 (Table 6). The other distinct amino acid of Agp2 is Arg83 (brown fields in Table 4 and Fig. 5B), which is notable for interacting with 5–6 Group 1 amino acids of Agp1—a unique feature because all other interactions involve only one or two amino acids. We previously reported [1] that among multiple *Agrobacterium* species, some contain both Agp1 and Agp2 homologs, and others only either an Agp1 or an Agp2 homolog. We selected six Agp2 homologs from species with two phytochromes and six Agp2 homologs from species with only one phytochrome and aligned their sequences. Arg83 is conserved in all Agp2 homologs from species that have two phytochromes, but only in three species out of six species with only one phytochrome (Fig. 6). This comparison could hint to a specific evolution of Arg83 for interaction with Agp1. Asn439, on the other hand (see above), is conserved in all 12 Agp2 homologs.

We note that several amino acids interact with two others of the partner protein (Table 6). We also inspected interacting amino acids located next to each other. One remarkable example is Arg545/Glu546 of Agp1 Group 1 that interact with Glu388/Arg390 of Agp2, Group 3. Their opposite charges could increase interaction strengths. Indeed, the distances at these positions are well below 2 Å.

### alphafold tetramer models

We also generated Agp1 and Agp2 interaction models by alphafold. This program was started with two Agp1 monomer and two Agp2 monomer sequences. Homodimer formation of Agp1 and Agp2 was not implemented as before, this makes other arrangements also possible. The 28 ranked Alphafold models were highly similar to each other, so we present the surface structure of model ‘ranked0’ in Fig. S1. In all alphafold models, the four subunits adopted a head‐to‐head arrangement with the appearance of a four‐leave clover when viewed from the N‐ or C‐terminal side. Each Agp1 or Agp2 monomer did not directly interact with the identical monomer but with the subunits of the other phytochrome. In the C‐terminal end, the four histidine kinases were tightly associated with each other. The N‐terminal parts were often more separate. The histidine kinase interaction of subunits within the Agp1 or Agp2 homodimer as proposed before and above was not realized in these models. In this respect, the alphafold tetramers differ from the docking models above. In all alphafold models, the calculated *E*_FRET_ value of the Position 122 was higher than the calculated *E*_FRET_ value of the Position 554, in contrast to the measurements (Table 4). In addition, the square of *E*_FRET_ differences for model ranked0 was always very high (Table 5). This makes the alphafold arrangements incompatible with our FRET results and confirms the assumption that interactions occur as two homodimers. Whereas for the formation of homodimers, alphafold generated suitable models, the calculation of Agp1 and Agp2 interaction did not. This in turn strengthens the above docking results.

## Discussion

The FRET studies have significantly enhanced our understanding of phytochrome–phytochrome interactions. By examining labeled Agp1 and Agp2 proteins, we confirmed their interaction and gained spatial insights through the use of various mutants. Specifically, FRET signals were detected in all six Agp1 mutants labeled at selected positions when mixed with Agp2 labeled at seven different positions. Truncated versions of Agp1 and Agp2 gave further spatial information about the interaction: No FRET signal was observed when labeled Agp1‐PCM was mixed with labeled Agp2, whereas a signal was present when labeled Agp2‐PCM was mixed with labeled Agp1.

The alphafold dimer models matches with existing PCM crystal structures of Agp1 and Agp2. The dimeric quaternary structure in parallel arrangement of monomer subunits is in line with two cryo‐EM full‐length structures of other bacterial phytochromes, PDB: ID 8UPK and PDB: ID 9EUT [6, 9]. Whereas the histidine kinases of 8UPK matches in its orientation with the Agp1 model (Fig. 1C), the orientation of the 9EUT histidine kinase is rotated 90° along the longitudinal axis. This means for our analyses that the Agp1 or Agp2 structures could differ from the alphafold model in this respect. Although we did not consider a rotated histidine kinase in our analyses, and the rotation would most likely not affect its outcome in general, the possibility for histidine kinase reorientation should be considered in ongoing studies.

Through the comparison of calculated *E*_FRET_ values from different docking models with the experimental data, three highly similar models emerged as the most plausible, while the others were excluded. According to these models, Agp1 and Agp2 dimers interact in such a way that Agp1 inserts its histidine kinase into a region of Agp2 between the PCM and histidine kinase, adjacent to the PCM, with their longitudinal axes oriented vertically. This finding lays the groundwork for further experimental studies based on site‐directed mutagenesis, to enhance the interaction between Agp1 and Agp2, potentially facilitating EM studies that require a strong interaction. Additionally, these docking models can serve as starting points for molecular dynamics studies.

In all selected models, Agp1 dimer and Agp2 dimer are oriented with their longitudinal axes at approximately right angles to each other. The interaction involves either subunit of Agp1 and Agp2. The line connecting both Agp1 histidine kinases is parallel to the longitudinal axis of the Agp2 dimer.

The evolution of an interaction could be based on interaction of existing groups of amino acids without extra adaptation. We believe that evolutionary most interesting amino acids are the separate ones that do not appear in groups. Arg83 of Agp2 is a good example for evolutionary adaptation. It is separate from other interaction amino acids on Agp2, and it interacts with 5–6 amino acids of Agp1, thus forming a tight connection between both partner proteins. A different amino acid at the position of Arg83 would probably affect the interaction significantly. The genus *Agrobacterium* contains many species, which can have either one or two phytochromes [1, 40]. We found that species with two phytochromes have Arg at Position 83, whereas those which have only Agp2 homologs can have other amino acids at that position. This correlation can give a clue to the evolution of the interaction.

The established interaction aligns with earlier observations that Agp2 reduces the autophosphorylation of Agp1 (Pr) by approximately 30%, while Agp1 has no effect on the Agp2 autophosphorylation [18]. The phosphor‐accepting residue in the Agp1 histidine kinase is located at Position 528, within Group 1. The positioning explains the inhibition of Agp1 autophosphorylation upon Agp2 binding and suggests that the interaction may be influenced by the phosphorylation state of Agp1. The negative charge of phosphorylated His528 of Agp1 would be approximately 4 Å away from Asp349 oxygen of Agp2. Agp1 also interacts with its cognate response regulator, which is transphosphorylated by the histidine kinase of Agp1. This interaction must involve the histidine kinase domain, as confirmed by a recent alphafold model [17]. While this interaction would interfere with the Agp1–Agp2 interaction on one side of the two Agp1 dimer subunits, proteins such as VirD2 or TraA could initiate signals of conjugation or plant gene transfer. VirD2 and TraA could bind to the histidine kinase region of Agp1 at angles distinct from that of the response regulator.

Studies of the Agp1/Agp2 interaction were prompted by evidence that both phytochromes function together in facilitating gene transfer to plants and bacterial conjugation [15, 16]. This suggests that Agp1 and Agp2 share initial steps in signal transduction. We propose that the Agp1–Agp2 interaction could either destabilize or stabilize the association of each phytochrome with VirD2, which initiates gene transfer, or TraA, which initiates conjugation, or with the response regulator. Such interactions could contribute to the complex regulation of these processes by light signals.

The insights gained from these FRET studies extend beyond Agp1 and Agp2 interactions, offering potential implications for other phytochromes in various organisms, including plants (but see the differing dimer structure of asymmetric monomer subunits [10]) and fungi, where such interactions have yet to be identified but are likely to occur. These findings could inspire biochemical and bioinformatics studies on other phytochromes to investigate their interactions and modes of interaction, broadening our understanding of phytochrome functions and mechanisms.

## Author contributions

AEK was involved in protein purification, labeling, and fluorescence measurements; GK and PS were involved in data evaluation; DH was involved in computer modeling and evaluation; EA was involved in computer modeling and evaluation, and figure design; ME was involved in coordination; NK was involved in data evaluation and writing; TL was involved in coordination, writing, data evaluation, and figure design.

### Peer review

The peer review history for this article is available at https://www.webofscience.com/api/gateway/wos/peer‐review/10.1002/1873‐3468.15102.

## Supporting information


**Fig. S1.** Agp1, and Agp2 interaction models.

## Data Availability

Data are available within the article or available upon request from the authors.
