# Supplementary material for: Interaction between bacterial phytochromes Agp1 and Agp2 of Agrobacterium fabrum by fluorescence resonance energy transfer and docking studies
Source: FEBS Lett. 2025 Jan 26;599(6):848–65. doi: 10.1002/1873-3468.15102 (PMC11931990; doi:10.1002/1873-3468.15102)
Supplement: Supplementary file 1 — Fig. S1. Agp1, and Agp2 interaction models. [file FEB2-599-848-s001.pdf]

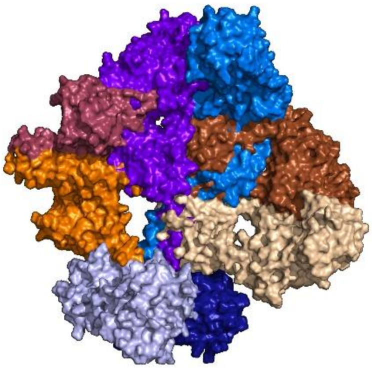

manual vertical

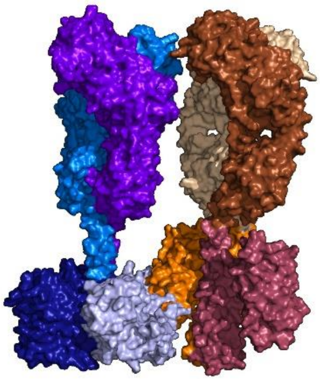

manual parallel

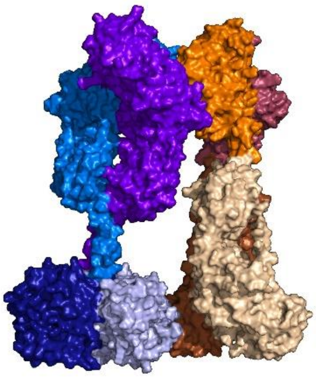

manual, antiparallel

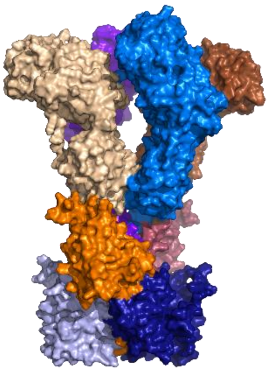

Alphafold, ranked1

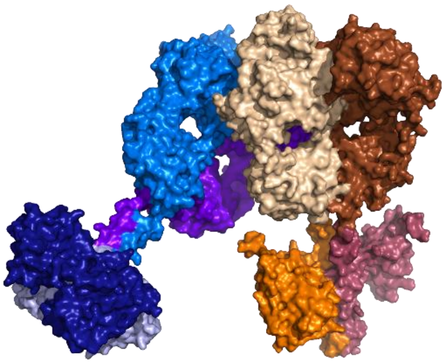

model.000.00

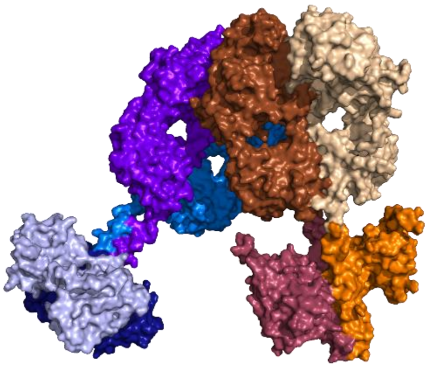

model000.01

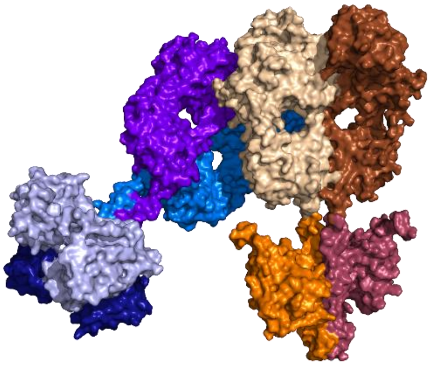

model000.02

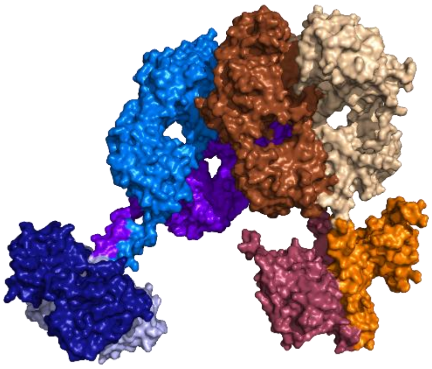

model000.03

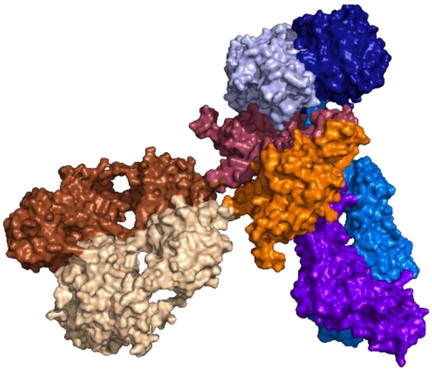

model000.04

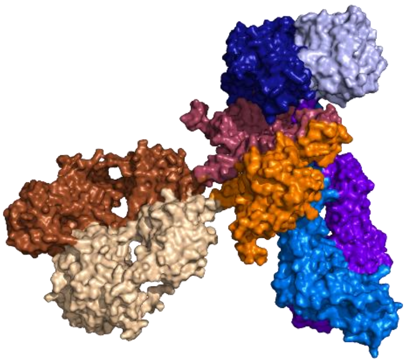

model000.05

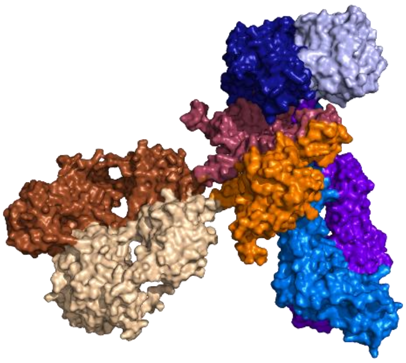

Model000.006

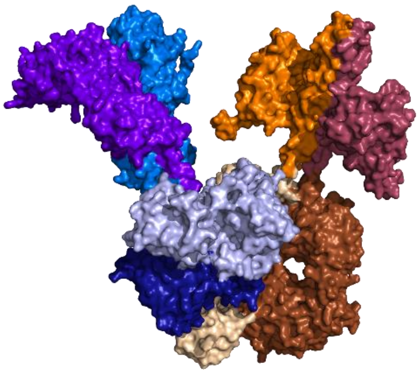

model000.07

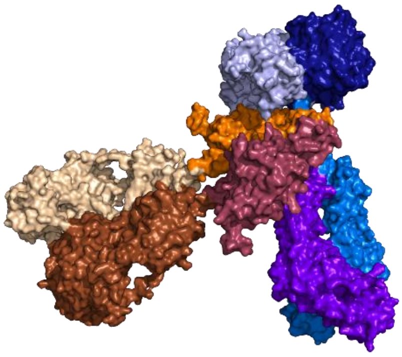

model000.08

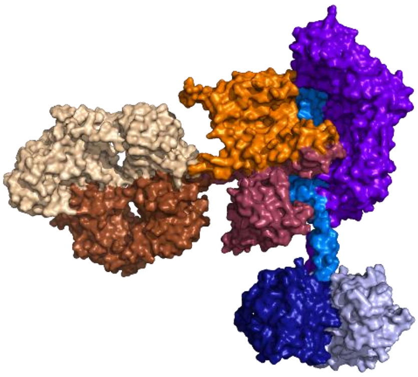

model000.09

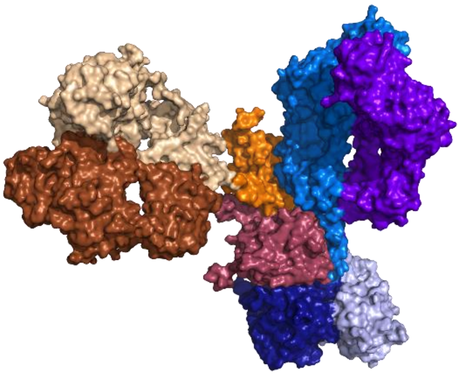

model002.00

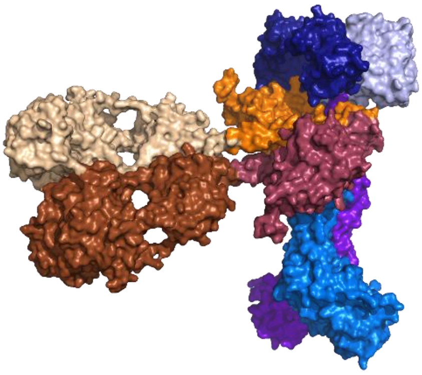

model002.01

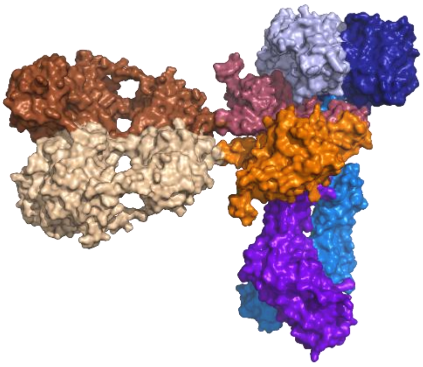

model002.02

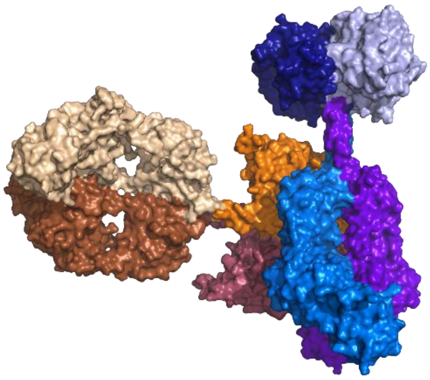

model002.03

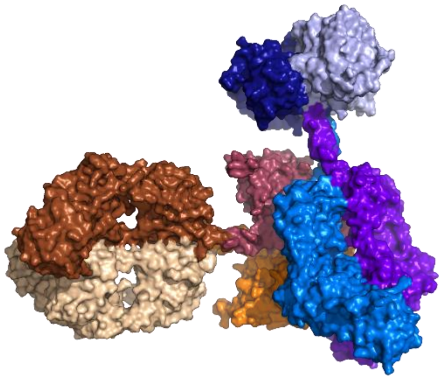

model002.04

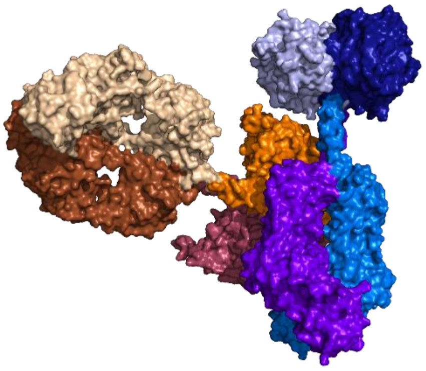

model002.05

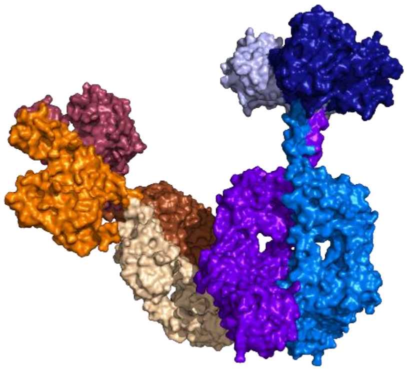

model002.06

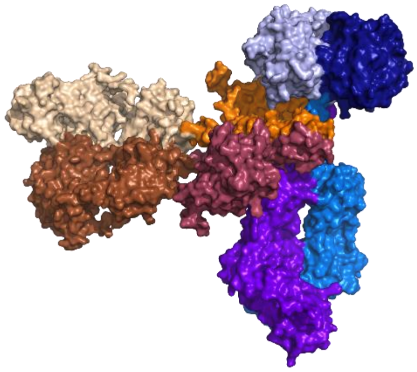

model002.07

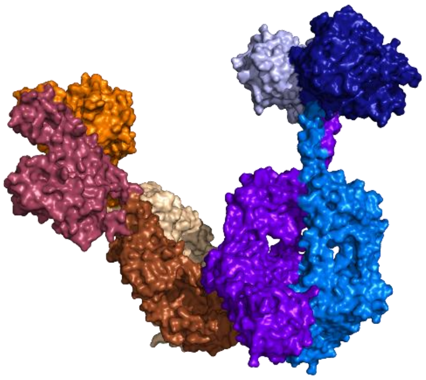

0model002.08

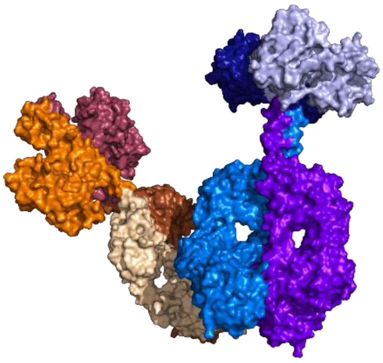

model002.09

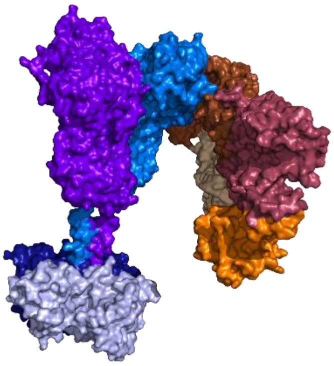

model004.00

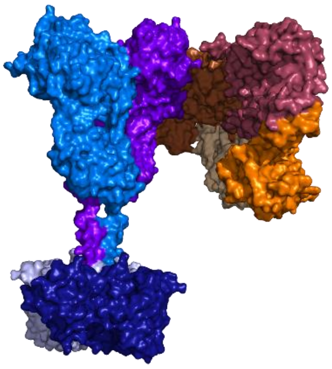

model004.01

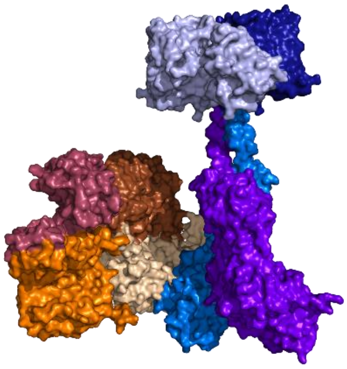

model004.02

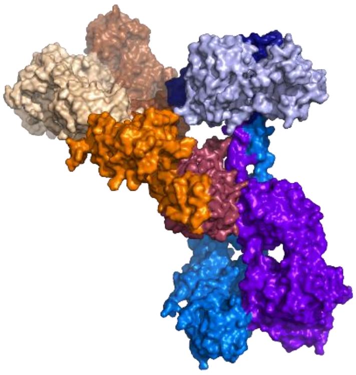

model004.03

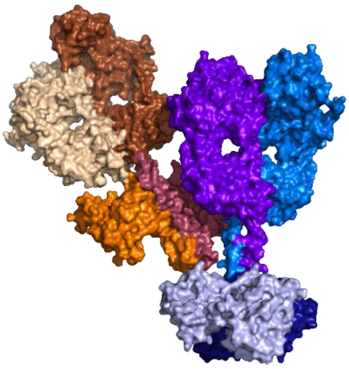

model004.04

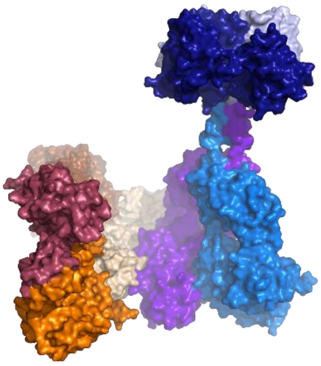

model004.05

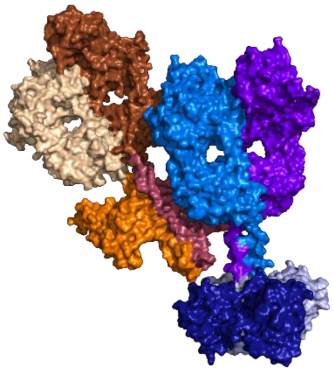

5

model004.06

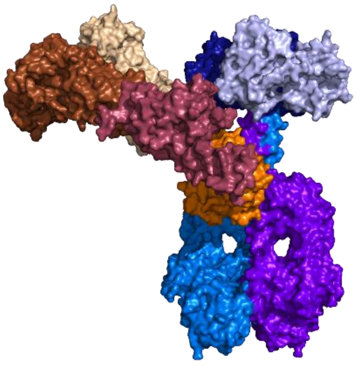

model004.07

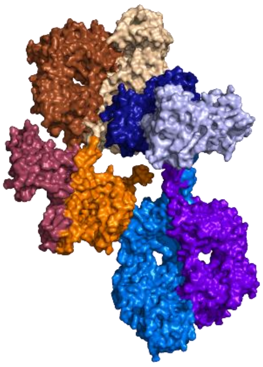

model004.08

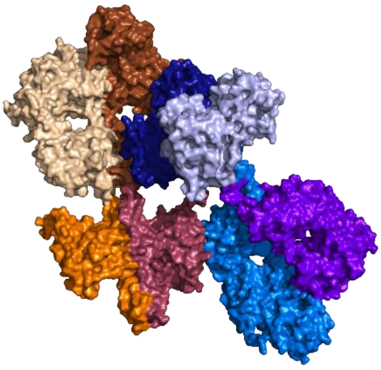

9

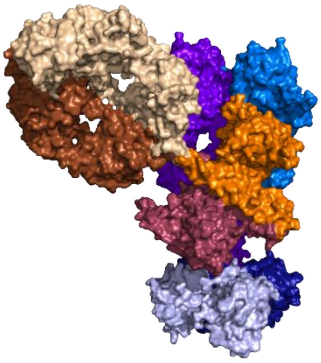

model006.00

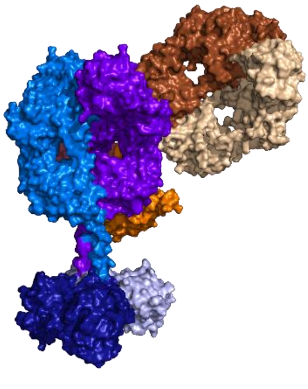

model006.01

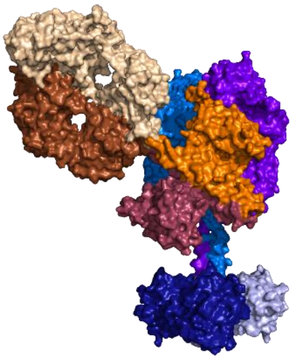

model004.02

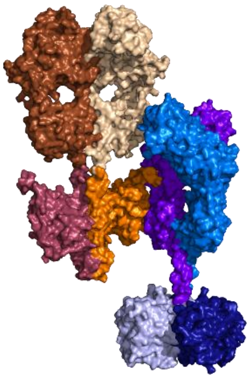

model004.03

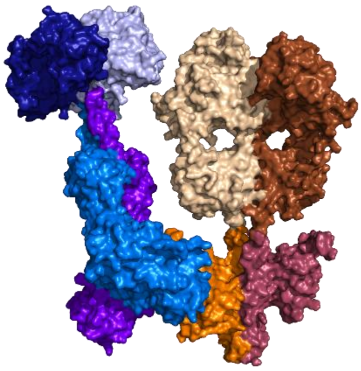

model004.04

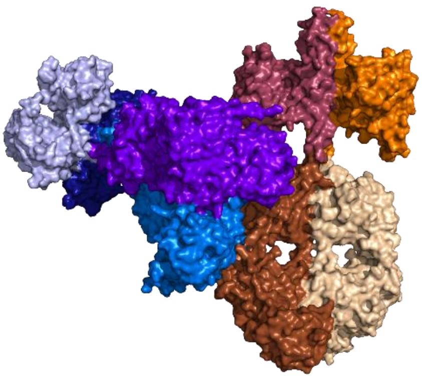

model004.05

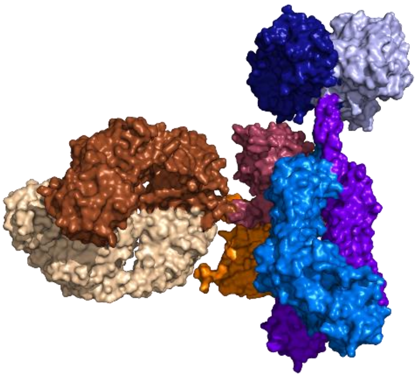

model004.06

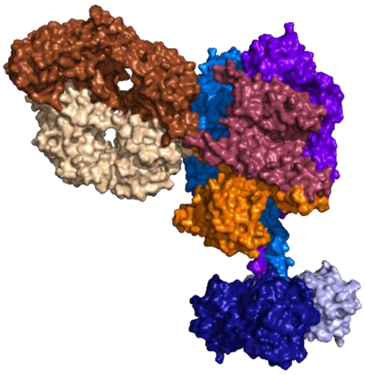

model004.07

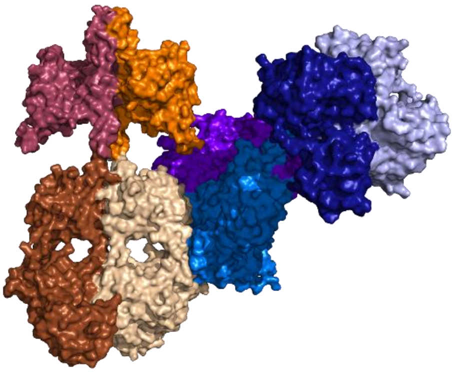

model004.08

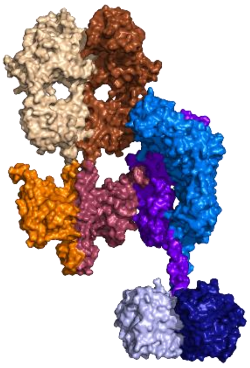

model004.09

SUPPLEMENTARY FIGURE S1.

Agp1 and Agp2 interaction models generated by Cluspro (model00x.xx), Alphafold (ranked0…) or manually, surface presentation. Agp1 subunits are colored in orange / brown tones, PCM domains by brown and beige, histidine kinases in orange and raspberry. Agp2 subunits are colored in blue / violet tones, PCM in purpleblue and blue, histidine kinases (including response regulator) in deep blue and light blue. The views in each panel are chosen to recognize the interaction regions.
